# Highly efficient C(CO)–C(alkyl) bond cleavage in ketones to access esters over ultrathin N-doped carbon nanosheets†

**DOI:** 10.1039/d2sc00579d

**Published:** 2022-04-11

**Authors:** Manli Hua, Jinliang Song, Xin Huang, Honglei Fan, Tianbin Wu, Qinglei Meng, Zhanrong Zhang, Buxing Han

**Affiliations:** Beijing National Laboratory for Molecular Sciences, CAS Key Laboratory of Colloid, Interface and Chemical Thermodynamics, CAS Research/Education Center for Excellence in Molecular Sciences, Institute of Chemistry, Chinese Academy of Sciences Beijing 100190 China hanbx@iccas.ac.cn; School of Chemistry Engineering, University of Chinese Academy of Sciences Beijing 100049 China; School of Chemical Engineering and Light Industry, Guangdong University of Technology Guangzhou 510006 China songjl_2021@gdut.edu.cn

## Abstract

Selective oxidative cleavage of the C(CO)–C bond in ketones to access esters is a highly attractive strategy for upgrading ketones. However, it remains a great challenge to realize this important transformation over heterogeneous metal-free catalysts. Herein, we designed a series of porous and ultrathin N-doped carbon nanosheets (denoted as CN-*X*, where *X* represents the pyrolysis temperature) as heterogeneous metal-free catalysts. It was observed that the fabricated CN-800 could efficiently catalyze the oxidative cleavage of the C(CO)–C bond in various ketones to generate the corresponding methyl esters at 130 °C without using any additional base. Detailed investigations revealed that the higher content and electron density of the graphitic-N species contributed to the excellent performance of CN-800. Besides, the high surface area, affording active sites that are more easily accessed, could also enhance the catalytic activity. Notably, the catalysts have great potential for practical applications because of some obvious advantages, such as low cost, neutral reaction conditions, heterogeneous nature, high efficiency, and broad ketone scope. To the best of our knowledge, this is the first work on efficient synthesis of methyl esters *via* oxidative esterification of ketones over heterogeneous metal-free catalysts.

## Abstract 2

Ultrathin and metal-free N-doped carbon nanosheets showed high activity and selectivity for oxidative esterification of ketones *via* C(CO)–C bond cleavage to access methyl esters.

## Graphical abstract

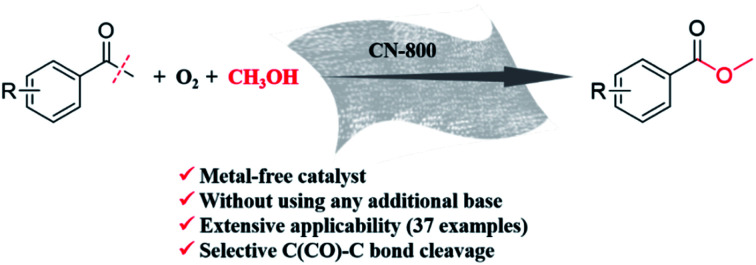

## Introduction

Ketones are a class of fundamental compounds with extraordinary versatility in the chemical community. Generally, ketones can be transformed into various value-added chemicals through the reconstruction of the C–H, C–O, or C(CO)–C bond.^1–7^ Molecular editing *via* the cleavage of the C(CO)–C bond is an emerging strategy to convert ketones into various value-added products, *e.g.*, esters, acids, nitriles, and amides.^8–15^ More specifically, the synthesis of methyl esters through the oxidative cleavage and esterification of the C(CO)–C bond in ketones has attracted increasing interest because methyl esters have diverse applications in solvents, polymers, synthetic intermediates, pharmaceuticals, *etc*.^16,17^ Notably, ketones can be abundantly obtained from renewable biomass.^18–22^ Thereby, from the importance of both methyl esters and biorefineries, it is a highly attractive strategy to synthesize methyl esters from the oxidative esterification of ketones *via* the C(CO)–C bond cleavage.

Initially, the synthesis of methyl esters from the oxidative esterification of ketones could be realized using stoichiometric oxidants, such as peroxides and metal salts.^23–25^ To make the processes more sustainable, O_2_ has been recognized as the ideal oxidant due to its features of abundant availability, naturality, and environmental benignity.^26^ To date, some catalytic systems have been developed for the aerobic oxidative esterification of ketones to synthesize methyl esters. For examples, the synthesis of methyl esters *via* aerobic oxidative esterification of ketones could be conducted by employing homogeneous copper (CuBr and CuCl_2_)^27–29^ or cerium salts as the catalysts.^30,31^ However, ligands and/or additives were essential in these catalytic systems,^7,14,17^ and the homogeneous feature made the reusability of these catalysts difficult. In comparison, heterogeneous catalysts are more desired, yet successful heterogeneous cases for aerobic oxidative esterification of ketones to synthesize methyl esters were very limited. To the best of our knowledge, only supported Zn^2+^ coordinated with N species on N-doped carbon could heterogeneously catalyze this transformation.^7^ Despite the fact that metal-based catalysts showed good activity in aerobic oxidative esterification of ketones, they suffered from several obvious drawbacks, including high cost, potential toxicity, poor durability, and metal contaminants (extremely unwanted in pharmaceuticals).^32,33^ To solve these drawbacks in metal-based catalytic systems, significant attention has been devoted to cost-effective metal-free catalytic systems, and several metal-free systems have been successfully developed for aerobic oxidative esterification of ketones to synthesize methyl esters.^4,34^ Nevertheless, these reported systems metal-free catalytic systems were all homogeneous, and needed special oxidants (*e.g.*, oxone) and catalysts (*e.g.*, NH_4_I)^34^ or strong bases (*e.g.*, KO^*t*^Bu) and special reactants (3-oxopropanenitrile).^4^ Undoubtedly, developing highly active, low-cost, and heterogeneous metal-free catalysts to realize the synthesis of methyl esters *via* aerobic oxidative esterification of ketones is highly desirable, but is a great challenge.

Carbon-based materials are a typical class of metal-free materials with distinct inherent characteristics, *e.g.*, abundant and even renewable resources (*e.g.*, lignocellulose) on earth, and low cost.^35–37^ Carbon materials have been successfully applied as efficient metal-free catalysts in diverse organic reactions,^38,39^ and their catalytic activity can be well adjusted by doping different heteroatoms (*e.g.*, N, S, Se, P, B),^40–44^ tuning the morphology from 0D carbon quantum dots to 3D porous bulk carbon, and even the synergism of these two strategies.^45–47^ However, the applications of metal-free carbon catalysts in catalysing aerobic oxidative esterification of ketones to access methyl esters have not been realized in view of the inherent kinetic inertness and thermodynamic stability of C(CO)–C bonds,^48,49^ and the limited catalytic activity of carbon catalysts in this transformation in comparison with that of metal-based catalysts. Much effort should be devoted to developing effective protocols to construct robust metal-free carbon catalysts to realize aerobic oxidative esterification of ketones *via* C(CO)–C bond cleavage to synthesize methyl esters.

In this work, we prepared porous N-doped carbon nanosheets (denoted as CN-*X*, where *X* represents the pyrolysis temperature) by pyrolyzing the mixture of chitosan (a renewable material) and melamine (a cheap chemical). It was observed that the synthesized CN-800 as a metal-free and heterogeneous catalyst showed outstanding catalytic activity in the aerobic oxidative esterification of ketones to synthesize methyl esters without using any basic additives, and various methyl esters could be efficiently and selectively obtained using these heterogeneous metal-free catalytic systems. To the best of our knowledge, this work for the first time offers an efficient, heterogeneous and metal-free catalyst for the synthesis of methyl esters *via* aerobic oxidative-cleavage of C(CO)–C bonds in ketones.

## Results and discussion

### Preparation and characterization of the catalysts

**Fig. 1:**
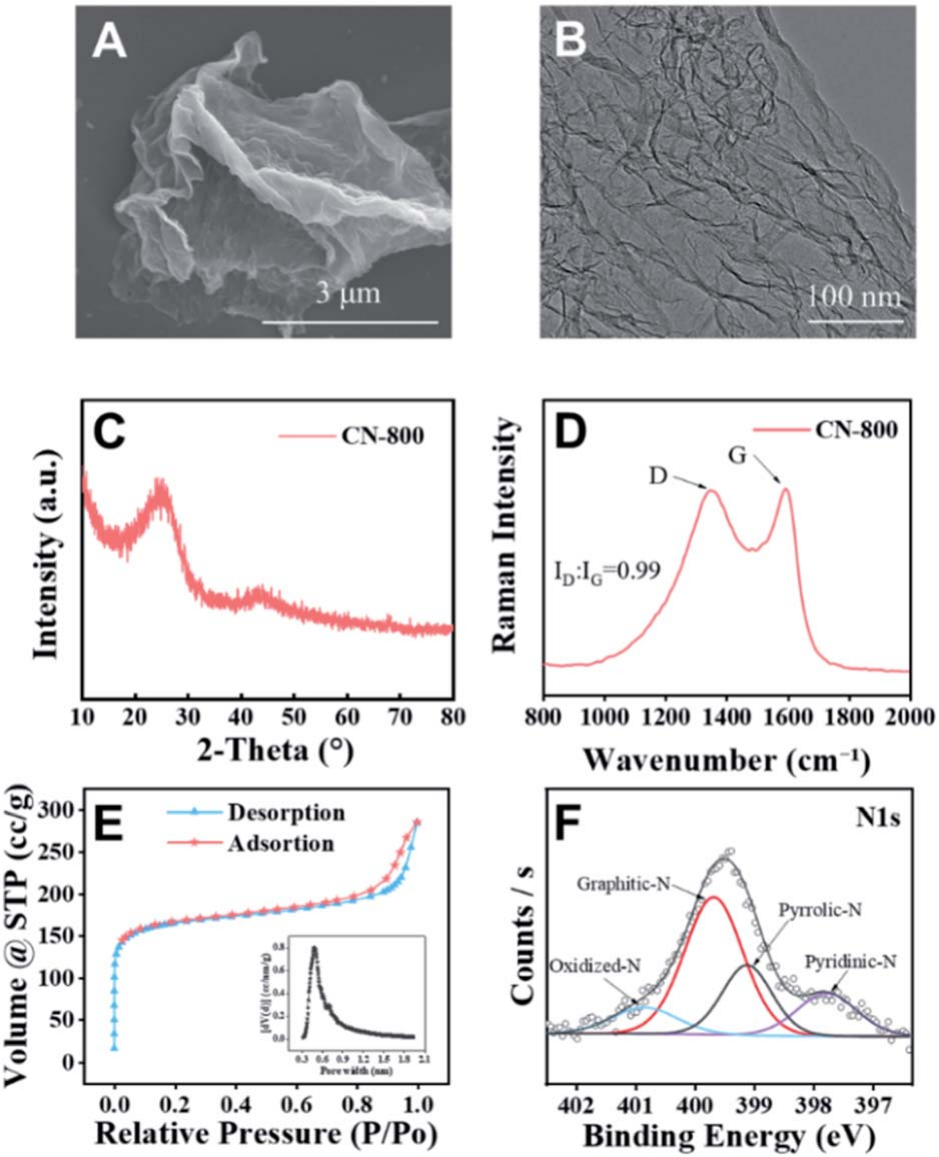
Characterization of the synthesized CN-800. (A) SEM image, (B) TEM image, (C) XRD pattern, (D) Raman spectrum, (E) N_2_ adsorption–desorption isotherm (the inset image in E: distribution of micropores), and (F) N 1s XPS spectra.

The desired CN-*X* catalysts were prepared by the pyrolysis of a highly-mixed mixture of chitosan and melamine, and the detailed procedure is discussed in the ESI.† CN-800 as the representative catalyst was characterized systematically. As detected by SEM (Fig. 1A), TEM (Fig. 1B) and HR-TEM (Fig. S1†), CN-800 had an obvious structure of ultrathin nanosheets with plenty of micropores, and the thickness of CN-800 was less than 1 nm based on AFM results (Fig. S2†). From the results of energy dispersive X-ray (EDX) mapping, it is found that the elements of C and N are distributed uniformly in the prepared CN-800 (Fig. S3†). The powder X-ray diffraction (XRD) pattern of CN-800 had two broad peaks at ∼25° and ∼44° (Fig. 1C), which corresponded to the (002) and (101) planes of the graphitic carbon. Meanwhile, obvious D-band and G-band, two characteristic bands of graphitic carbon materials, were observed in the Raman spectrum of CN-800 (Fig. 1D), and many structural defects existed in CN-800 as deduced from the intensity ratio of D/G bands. As determined by the N_2_ adsorption–desorption technique, the CN-800 exhibited a well-defined microporous structure with the micropore size centered at 0.51 nm (Fig. 1E), which was consistent with the HR-TEM result, and the BET surface area of CN-800 was 631 m^2^ g^−1^ (Table S1†). Furthermore, the presence of C, N, and O was confirmed by the XPS survey spectrum (Fig. S4†). It can be known from the high resolution XPS spectrum of N 1s (Fig. 1F) that four types of N species, including pyridine-type N (397.77 eV), pyrrole-type N (399.11 eV), graphitic-type N (399.68 eV), and oxidized N (400.85 eV), were clearly observed. Additionally, the content of N in CN-800 was 8.72 wt% as determined by element analysis (Table S2†).

### Catalytic activity of various metal-free catalysts

The catalytic performance of the prepared metal-free CN-*X* catalysts was evaluated by employing oxidative conversion of acetophenone into methyl benzoate as a model reaction (Table 1). The reaction could not proceed in the absence of a catalyst (Table 1, entry 1). To our delight, the prepared CN-800 showed excellent catalytic activity for the reaction (Table 1, entry 2), and a quantitative yield of the target product was achieved. In comparison, several other carbon-based catalysts were prepared by varying the weight ratio of chitosan and melamine, and the pyrolysis temperature. As can be known from the results of TEM (Fig. S5†), XRD (Fig. S6†), Raman spectroscopy (Fig. S7†), N_2_ adsorption–desorption (Table S1†), and element analysis (Table S2†), the morphology and the properties of the fabricated materials were significantly affected by the preparation parameters, thereby resulting in their different catalytic activities. For example, the pyrolysis temperature has a great influence on the reactivity (Table 1, entries 2–5), and the catalytic activity of the obtained catalysts increased with increasing the pyrolysis temperature from 500 to 800 °C, among which CN-800 possessed the highest activity while CN-500 showed very poor activity. Similarly, the catalytic activity increased with increasing the ratio of chitosan and melamine (Table 1, entries 2, 6 and 7), and increasing the usage of chitosan could enhance the catalytic activity. However, CN-Chit, prepared using pure chitosan at 800 °C, showed low catalytic activity (Table 1, entry 8). Additionally, no reaction occurred over CN-Cell (Table 1, entry 9), which was fabricated using cellulose as the raw material. The results above indicated that CN-800 was the best catalyst among the prepared metal-free catalysts, and the doping of N played a crucial role in achieving high activity for the studied reaction.

**Table 1:** Catalytic activity of various catalysts for oxidative conversion of acetophenone into methyl benzoate^a^

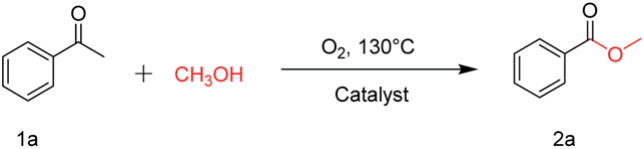			
Entry	Catalysts	Conversion^b^ (%)	Yield^b^ (%)
1	None	N.D.	N.D.
2	CN-800	>99	>99
3	CN-500	N.D.	N.D.
4	CN-600	38	25
5	CN-700	79	75
6^c^	CN-1 : 6	73	67
7^d^	CN-1 : 3	88	81
8^e^	CN-Chit	16	16
9^f^	CN-Cell	N.D.	N.D.
10^g^	CN-800	N.D.	N.D.

a Reaction conditions: acetophenone, 0.5 mmol; ethylbenzene, 0.5 mmol; catalyst, 100 mg; MeOH, 3 mL; O_2_, 5 bar; reaction temperature, 130 °C; reaction time, 10 h.

b The conversion and yield were determined by GC using ethylbenzene as an internal standard.

c The catalyst was prepared using a chitosan/melamine weight ratio of 1 : 6 at 800 °C.

d The catalyst was prepared using a chitosan/melamine weight ratio of 1 : 3 at 800 °C.

e The catalyst was prepared using pure chitosan at 800 °C.

f The catalyst was prepared using pure cellulose at 800 °C.

g Ar (5 bar) was used to replace O_2_.

### Optimization of reaction conditions

**Fig. 2:**
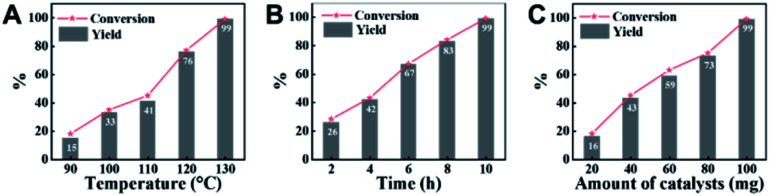
Influence of various reaction parameters. (A) Reaction temperature, (B) reaction time, and (C) amount of CN-800. Reaction conditions: acetophenone, 0.5 mmol; ethylbenzene, 0.5 mmol; MeOH, 3 mL; 5 bar O_2_; reaction temperature, 130 °C for (B and C); reaction time, 10 h for (A and C); usage of CN-800, 100 mg for (A and B). The conversions and yields were determined by GC using ethylbenzene as an internal standard.

Employing CN-800 as the catalyst, various reaction parameters were subsequently optimized. As expected, the conversion of acetophenone increased when the reaction temperature changed from 90 to 130 °C (Fig. 2A). The reaction could be completed at 130 °C with a reaction time of 10 h (Fig. 2B), and the yield of the target methyl benzoate could reach 99%. Furthermore, it was found that the usage of CN-800 also had significant impact on the reaction efficiency (Fig. 2C), and the conversion of acetophenone and the yield of methyl benzoate gradually increased when the catalyst usage increased from 20 to 100 mg. With a CN-800 usage of 100 mg, acetophenone could be converted to methyl benzoate with a yield of 99%. Based on the experimental results, we can conclude that the optimal reaction conditions were 130 °C, 10 h, and 100 mg CN-800. Besides, when the reaction was conducted under an Ar atmosphere (Table 1, entry 10), no reaction occurred, suggesting that O_2_ was essential in this transformation. However, the influence of O_2_ pressure on the reaction efficiency was not significant in the range of 1 to 5 bar (Table S3†), indicating that the O_2_ pressure was not the determinant for the reaction activity in the examined pressure range.

### Reusability of CN-800

**Fig. 3:**
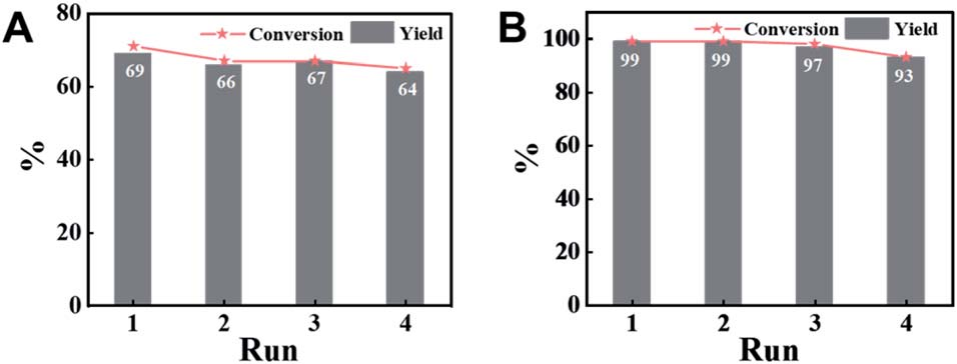
Recyclability of CN-800. Reaction conditions: acetophenone, 0.5 mmol; ethylbenzene, 0.5 mmol; CN-800, 100 mg; MeOH, 3 mL; 5 bar O_2_; 130 °C; 6 h for (A) and 10 h for (B). Yield was determined by GC using ethylbenzene as an internal standard.

An attractive merit of heterogeneous catalysts is their capability to be recycled. Thus, the reusability of CN-800 was evaluated by using acetophenone as the substrate. There were no considerable changes in both the conversion of acetophenone and the yield of methyl benzoate in four consecutive cycles with a reaction time of 6 h (Fig. 3A) or 10 h (Fig. 3B) when the original usage of CN-800 was 100 mg. Moreover, no obvious difference was found between the fresh CN-800 and the recovered one, as characterized by various techniques, including TEM (Fig. S8†), Raman spectroscopy (Fig. S9A†), XRD (Fig. S9B†), N_2_ adsorption–desorption (Fig. S9C†), XPS (Fig. S9D†), and elemental analysis (Table S2†). In addition, CN-800 could still be well recycled for four catalytic cycles (Fig. S10A†) when the usage of CN-800 was changed to 60 mg. The results above verified the good stability and reusability of CN-800 under the reaction conditions. In another respect, the heterogeneous nature of CN-800 was evaluated by a control experiment (Fig. S10B†), in which the CN-800 was removed from the catalytic system when the reaction was proceeded for 4 h, and then the reaction was continually conducted for 6 h without the solid catalyst. No further reaction was observed after the removal of CN-800, confirming the negligible leaching of active species and the heterogeneous nature of CN-800.

### Scope of substrates

Inspired by its excellent catalytic performance in the conversion of acetophenone, the applicability of CN-800 to the oxidative esterification of other (het) aryl methyl ketones was investigated (Table 2). It was found that various substituted acetophenones could be selectively converted into the corresponding methyl esters with satisfactory yields. Meanwhile, the properties of the substituents showed weak impact on the reactivity of the substrates. No matter electron-withdrawing (1b–1f, including –CF_3_, –CN, –F, –Cl, –Br) or electron donating (1g–1i, including –CH_3_, –OCH_3_, –OCH_2_CH_3_) substituents, excellent yields (>90%) of methyl esters could be obtained when they were at the *para*-position. In contrast, the position of the substituents could significantly affect the reactivity, and the *para*-substituted substrate showed the highest reactivity while the *ortho*-substituted substrates had the poorest reactivity. For example, the reactivity of Br-substituted acetophenones decreased as follows: *para*-bromoacetophenone (1f) > *meta*-bromoacetophenone (1j) > *ortho*-bromoacetophenone (1k). Similarly, the reactivity of Me-substituted acetophenones (1g, 1l, 1m) followed the same tendency. Moreover, the reaction efficiency decreased with the increasing number of substituents. For instance, among MeO-substituted acetophenones (1h, 1n–1p), the reactivity of the *mono*-substituted substrate was highest, while the tri-substituted substrate showed the lowest reactivity. Additionally, 1-acetonaphthone (1q) showed lower activity than acetophenone (1a). The results above were probably caused by the steric resistance: both *ortho*-substituents (1k, 1m) and multi-substituents (1n–1p, 1r, 1s) increased the steric resistance of the corresponding substrates. Besides, phenyl- and phenoxy-substituted acetophenones (1t, 1u) could also be efficiently converted into the corresponding methyl esters over CN-800 under standard reaction conditions. Notably, several challenging heteroaryl methyl ketones, *i.e.*, 2-acetylfuran (1v), 2-acetylthiophene (1w), and 4-acetylpyridine (1x), could also be efficiently oxidized into the desired methyl esters with yields of >90%, further confirming the robust activity of CN-800 for the reactions.

**Table 2:** Substrate scope of phenyl substituted ketone over CN-800

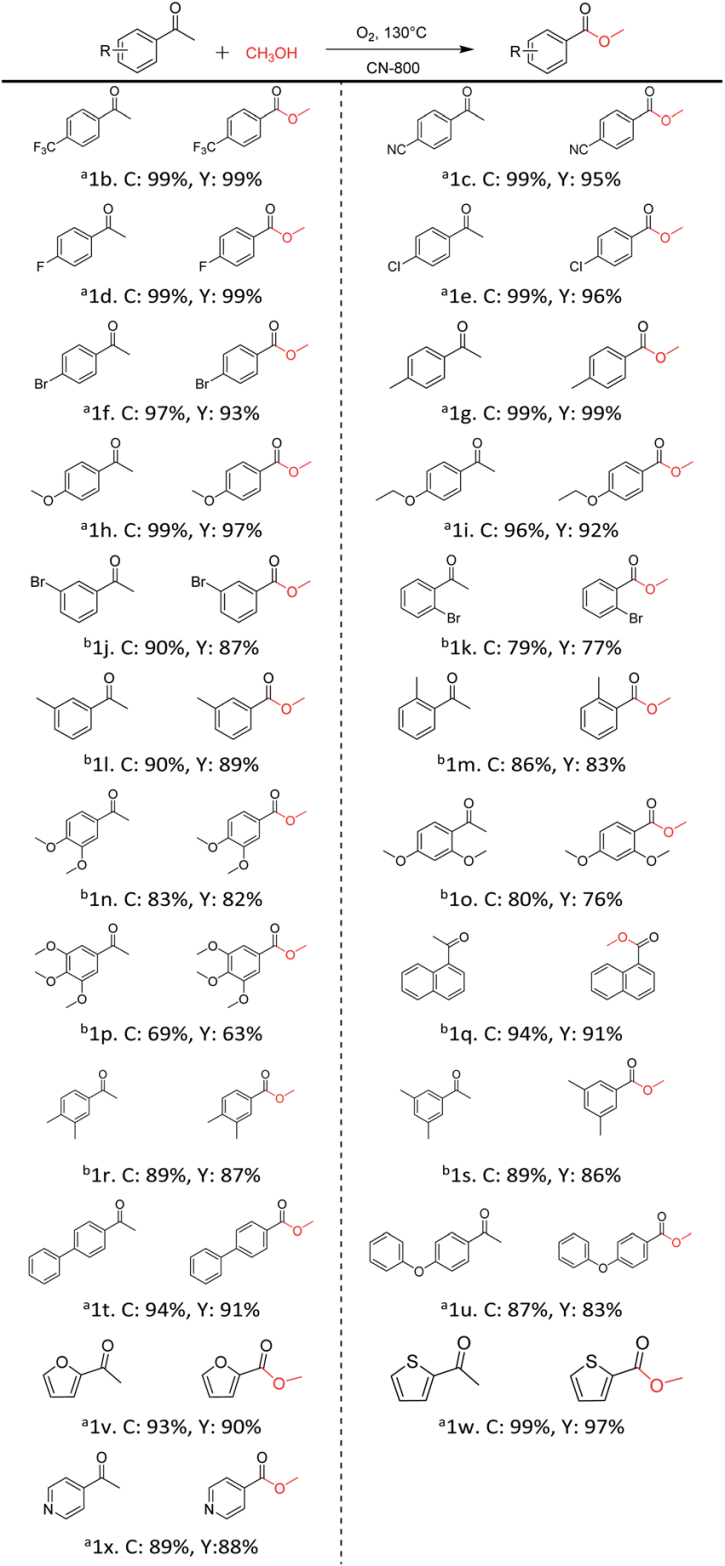

a Reaction conditions: substrate, 0.5 mmol; ethylbenzene, 0.5 mmol; CN-800, 100 mg; methanol, 3 mL; 5 bar O_2_; 130 °C; 10 h.

b Reaction conditions: substrate, 0.5 mmol; ethylbenzene, 0.5 mmol; CN-800, 100 mg; methanol, 3 mL; 5 bar O_2_; 130 °C; 24 h. Yield was determined by GC using ethylbenzene as an internal standard.

In addition, the influence of different aliphatic alcohols (ethanol, *n*-propanol, and *n*-butanol) was investigated. The results indicated that an additional base (*e.g.*, K_2_CO_3_) was essential to facilitate the oxidative conversion of acetophenone into the corresponding esters with these three alcohols (Table S4†). Moreover, the reactivity of the alcohols decreased with the increase of the carbon chain length of the alcohols, which was consistent with some reported results.^7^ These results probably resulted from that the dissociation energy of the O–H bond and the steric hindrance increased with the increase of the alkyl chain. Thus, further optimization was required to make this CN-800 catalytic system effective for various alcohols.

Generally, acetophenones substituted at the β-C position have lower reactivity, thereby being more challenging to be converted into the corresponding esters *via* oxidative esterification. Herein, the reactivities of several acetophenones substituted at the β-C position were examined over the CN-800. Obviously, the C(CO)–C bonds in these acetophenones could be smoothly converted into the corresponding methyl benzoates with excellent yields of >90% (Table 3, entries 1–7). When there were more substituents at the β-C position (Table 3, entry 8), the reactivity decreased remarkably with the increasing number of substituents. Additionally, when the substituents were at both the β-C position and phenyl group, the reactivity also decreased (Table 3, entries 9 and 10), and a prolonged reaction time (24 h) was needed to achieve a satisfactory product yield. Moreover, 2-phenoxyacetophenone, an important lignin model, could also be efficiently converted to provide methyl benzoate in a yield of 93% (Table 3, entry 11). Surprisingly, 1,3-indandione (Table 3, entry 12) and 1-indanone (Table 3, entry 13) could be oxidized, and phthalic anhydride was generated as the main product. These results further verified the superior catalytic performance of CN-800 in the aerobic oxidative esterification of ketones *via* C(CO)–C bond cleavage to synthesize methyl esters. Unfortunately, CN-800 showed very low or even no activity in the transformation of alkyl ketones (*e.g.*, 4-phenyl-2-butanone, α-ionone, 3-hepten-2-one, 2-octanone, and 2,5-hexanedione) to produce the desired esters (Table 3, entries 14–18). Further construction of more robust metal-free carbon-based catalytic materials is required to realize the conversion of alkyl ketones into the corresponding esters *via* C(CO)–C bond cleavage.

**Table 3:** Aerobic oxidative esterification of more challenging ketones over CN-800^a^

Entry	Substrate	Product	Yield^b^ (%)
1	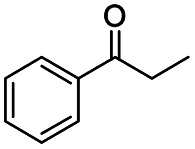	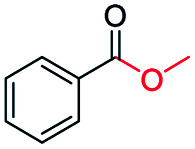	99
2	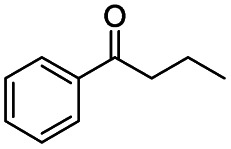	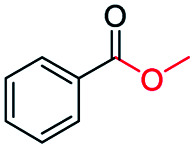	99
3	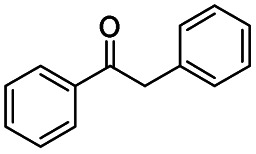	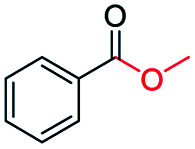	91
4	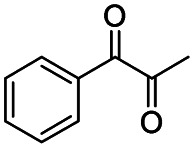	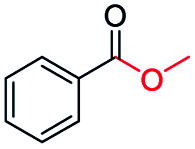	93
5	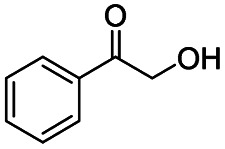	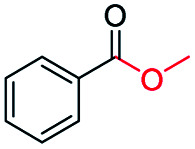	99
6	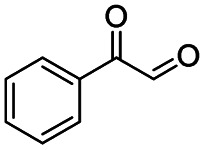	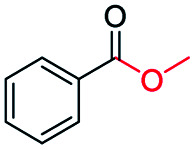	99
7	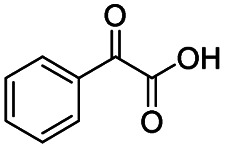	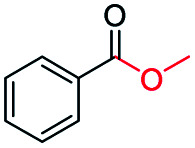	99
8	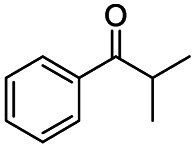	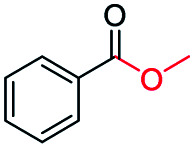	63 (82^c^)
9	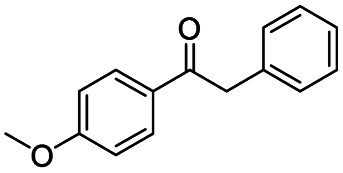	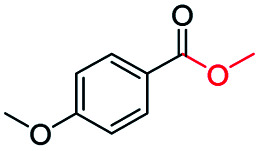	75^c^
10	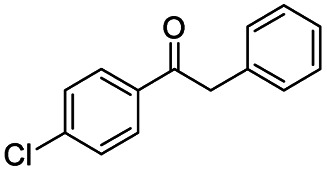	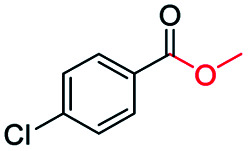	87^c^
11	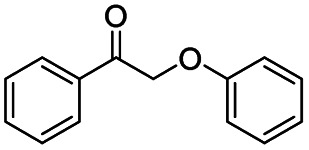	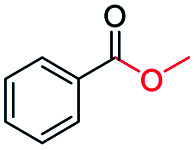	78
12	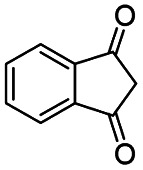	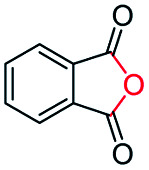	86^c^
13	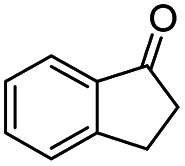	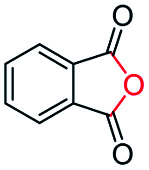	71^c^
14	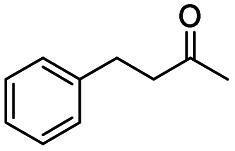	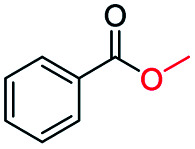	4
15	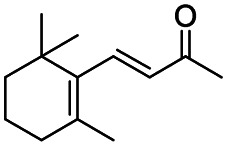	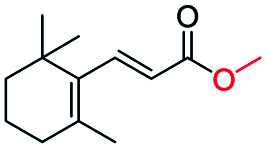	Trace
16	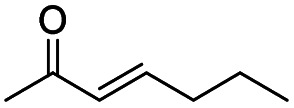	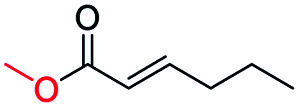	0
17	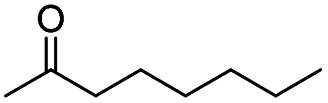	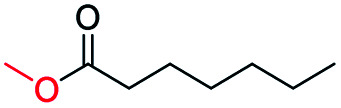	0
18	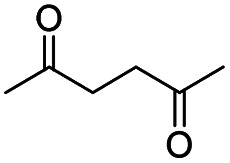	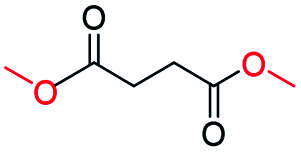	0

a Reaction conditions: substrate, 0.5 mmol; ethylbenzene, 0.5 mmol; CN-800, 100 mg; methanol, 3 mL; 5 bar O_2_; 130 °C; 10 h.

b Yield was determined by GC using ethylbenzene as an internal standard.

c Substrate, 0.5 mmol; ethylbenzene, 0.5 mmol; CN-800, 100 mg; methanol, 3 mL; 5 bar O_2_; 130 °C; 24 h.

### Analysis of catalytically active sites

It is of crucial importance to reveal the catalytically active sites and meanwhile discuss the reason for the superior performance of the CN-800. As is well known, graphitic-N species, the defects, and even sp_2_ carbon can be the potential catalytic sites in N-doped carbon materials. First, as characterized by Raman spectra, the intensity ratio of D and G bands in the prepared catalytic materials decreased when the pyrolysis temperature increased from 600 to 800 °C or the ratio of chitosan and melamine increased from 1 : 6 to 1 : 3 (Fig. S11†), indicating that the defects in the corresponding materials decreased with increasing pyrolysis temperature or ratio of chitosan and melamine. However, the catalytic activity of the prepared catalysts followed an opposite trend (Fig. S11†). Therefore, the defects were not the decisive catalytic sites in the prepared carbon-based catalysts. Second, CN-Chit had more sp^2^ carbon than other N-doped catalysts (Table S5 and Fig. S12†), but its catalytic activity was poorer than other N-doped catalysts except for CN-500 (Table 1). Meanwhile, CN-500 nearly showed no activity although it contained sp^2^ carbon. Thereby, it could be deduced that the catalytic role of the sp^2^ carbon could be excluded.

**Fig. 4:**
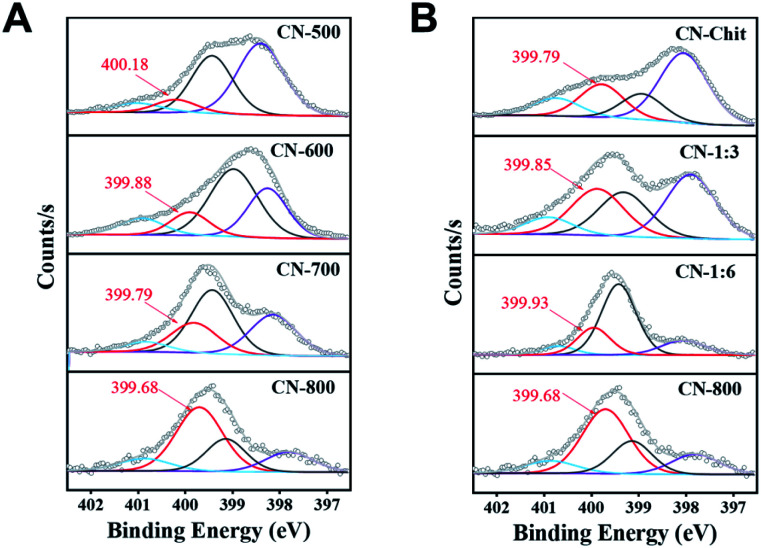
N 1s XPS spectra. (A) The catalysts prepared at different temperatures, and (B) the catalysts prepared with different molar ratios of chitosan and melamine.

From the catalytic results in Table 1, CN-Cell showed no catalytic activity, while CN-Chit could catalyze the reaction. The difference between CN-Cell and CN-Chit was that CN-Chit contained N species, while there was no doped-N in CN-Cell, indicating that the N species were the probable active sites for the reactions. It is widely accepted that the graphitic-N species are the catalytic sites in metal-free N-doped carbon catalysts.^50–55^ Based on the XPS results (Fig. 4), it was obvious that the content of graphitic-N species in the prepared materials was positively correlated with both the preparation temperature and the molar ratio of chitosan and melamine. Notably, the relative content of graphitic-N species increased in the order: CN-500 < CN-Chit < CN-600 < CN-1 : 6 < CN-700 < CN-1 : 3 < CN-800, which was consistent with their catalytic activity (Fig. S13† and Table 1), confirming that graphitic-N species were the catalytic sites in the prepared catalysts for the studied reactions. Importantly, the graphitic-N in CN-800 showed the lowest binding energy among all the examined catalysts, indicating that the graphitic-N in CN-800 possessed the highest electron density. Thus, the basicity of graphitic-N in CN-800 was the strongest, which was helpful for the electronic interaction of oxygen with graphitic-N species to generate more active oxygen species for promoting the reaction.^56^ Meanwhile, the reactivity of ketones for this transformation originated from that the existence of carbonyl groups could acidify the β-C_sp^3^_–H. However, the extent of acidification was too low. Thus, a catalyst was needed to increase the acidification of the β-C_sp^3^_–H.^7^ The graphitic-N with higher electron density could enhance the acidification of the β-C_sp^3^_–H originating from the carbonyl groups, thus promoting the abstraction of β-C_sp^3^_–H, which was beneficial for the reaction. Therefore, CN-800, having more content of more negatively charged graphitic-N species, provided the highest catalytic activity in the oxidative conversion of ketones into esters.

Besides, as characterized by the N_2_ adsorption–desorption method, the BET surface area of the prepared catalysts increased with the increase of the preparation temperature and the molar ratio of chitosan and melamine (Table S1†). CN-800 had a BET surface area of 631 m^2^ g^−1^, which was the highest among all the prepared catalysts. The high surface area afforded catalytically active sites that are more exposed and more easily accessed, thereby resulting in the highest catalytic activity of CN-800. CN-500 showed a very low surface area (14 m^2^ g^−1^), which made the active sites embedded in the bulk body and thus difficult to access. This was the probable reason for the ignorable catalytic activity of CN-500 although it contained a certain number of graphitic-N species. Additionally, carbon materials having high surface area were beneficial for the activation of gas (*e.g.*, O_2_, CO_2_) and organic molecules. Thus, CN-800 with the highest surface area had the strongest ability to activate O_2_ and the acetophenones, thereby showing the best catalytic performance.

From the discussion above, it can be known that the graphitic-N species were the catalytically active sites to catalyze the conversion of ketones into the corresponding esters *via* C(CO)–C(alkyl) bond cleavage, and their contents and electronic state significantly affected the catalytic activity. CN-800 had the most graphitic-N species, providing it with the most active sites. Moreover, the graphitic-N species in CN-800 were more negatively charged, making it have the strongest ability to enhance the acidification of the β-C_sp^3^_–H bond originating from the carbonyl group in ketones, and promote the formation of active oxygen species. These features enabled CN-800 to show the best catalytic performance. Additionally, owing to the highest surface area, the graphitic-N species in CN-800 was more exposed and more easily accessed. Meanwhile, the high surface area favored the activation of the reactants (*i.e.*, O_2_ and the acetophenones), and mass transfer, further improving the catalytic activity of CN-800. Therefore, it could be deduced that the excellent performance of CN-800 originated from the synergistic effect of graphitic-N species with higher electron density and the high surface area.

### Investigation of the reaction mechanism

**Scheme 1:**
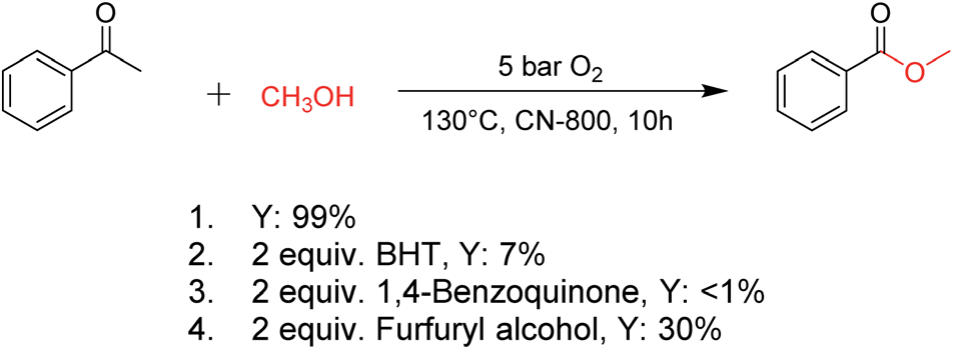
Control experiments with different radical scavengers. Reaction conditions: acetophenone, 0.5 mmol; ethylbenzene, 0.5 mmol; CN-800, 100 mg; methanol, 3 mL; O_2_, 5 bar; 130 °C; 10 h; the additive was added as above.

For oxidations involving C–H bond transformation, the reactions generally proceed through a radical pathway. To confirm this, several radical inhibition experiments were initially conducted (Scheme 1). The conversion of acetophenone into methyl benzoate was significantly inhibited after adding 2 equiv. 2,6-ditertbutyl-4-methylphenol (BHT, a radical scavenger), indicating that the reaction occurred *via* a radical reaction process. Furthermore, the addition of 2 equiv. benzoquinone (a O_2_˙^−^ radical scavenger) could completely quench the reaction, implying that O_2_˙^−^ radicals were the radical species to promote the oxidation. Meanwhile, the reaction was partially quenched when 2 equiv. of furfuryl alcohol (a singlet oxygen scavenger) was added into the reaction system, indicating the formation of singlet oxygen from the O_2_˙^−^ radicals in the reaction process. These results confirmed that the studied oxidations were promoted by O_2_˙^−^ radicals.

Subsequently, the reaction pathway was investigated. First, as discussed above, the reactivity would decrease when there was more than one substituent on the β-C position. For example, propiophenone had similar reactivity to acetophenone, while the yield of methyl benzoate was only 63% when using isobutyrophenone as the substrate. In particular, no methyl benzoate was generated from 2,2-dimethylpropiophenone (Scheme S1a†). These results indicated that at least one H at the β-C position was essential. Second, very low yields (4% and 6%) of methyl benzoate were achieved from benzoic acid and benzaldehyde, respectively (Scheme S1b†), indicating that the reaction did not occur through the pathway of forming benzoic acid and benzaldehyde. Third, an obvious proton/deuterium kinetic isotope effect was observed when using acetophenone-*d*_3_ as the substrate (Scheme S2†), implying that the oxidation of the β-C_sp^3^_–H bond probably was the rate-determining step. Meanwhile, 2-hydroxyacetophenone, 2-oxo-2-phenylacetaldehyde, and benzoylformic acid showed much higher reactivity than acetophenone (Scheme S3†), and 2 h was enough to completely convert these substrates into methyl benzoate (99% yield), suggesting that 2-hydroxyacetophenone, 2-oxo-2-phenylacetaldehyde, and benzoylformic acid were the potential intermediates. Furthermore, 2 equiv. benzoquinone could completely quench the conversion of 2-hydroxyacetophenone, while the addition of 2 equiv. benzoquinone nearly showed no impact on the conversion of 2-oxo-2-phenylacetaldehyde and benzoylformic acid (Scheme S3†). In comparison, when the conversion of 2-hydroxyacetophenone, 2-oxo-2-phenylacetaldehyde and benzoylformic acid was conducted under an Ar atmosphere (5 bar), benzoylformic acid could be completely converted, while the conversion of 2-oxo-2-phenylacetaldehyde was only 37%, and nearly no reaction occurred with 2-hydroxyacetophenone as the reactant (Scheme S3†). These results indicated that to obtain methyl benzoate, further radical oxidation was required for 2-hydroxyacetophenone, while 2-oxo-2-phenylacetaldehyde could be converted through two reaction pathways, including (1) being directly converted into methyl benzoate without the need to be further oxidized, and (2) being firstly oxidized into benzoylformic acid *via* a non-radical process and subsequently being converted into the desired methyl benzoate (the main pathway considering the conversion of phenylacetaldehyde under an Ar atmosphere). Thereby, the successive oxidation of acetophenone into 2-hydroxyacetophenone and 2-oxo-2-phenylacetaldehyde (as two intermediates) could be deduced as a probable reaction, while whether further oxidation of 2-oxo-2-phenylacetaldehyde into benzoylformic acid or nor relied on the type of substrate (see the description of the mechanism). Finally, 2 equiv. of dimethyl sulfoxide (a HO˙ radical scavenger) could partially quench the reaction (Scheme S4†), implying that the generation of R–O˙ and HO˙ radicals *via* the homolytic rupture of the *in situ* formed R–O–O–H was another potential reaction pathway, and the substrates with only one H at the β-C position (*e.g.*, isobutyrophenone and cyclopentyl phenyl ketone) were oxidized only through this reaction pathway.

**Scheme 2:**
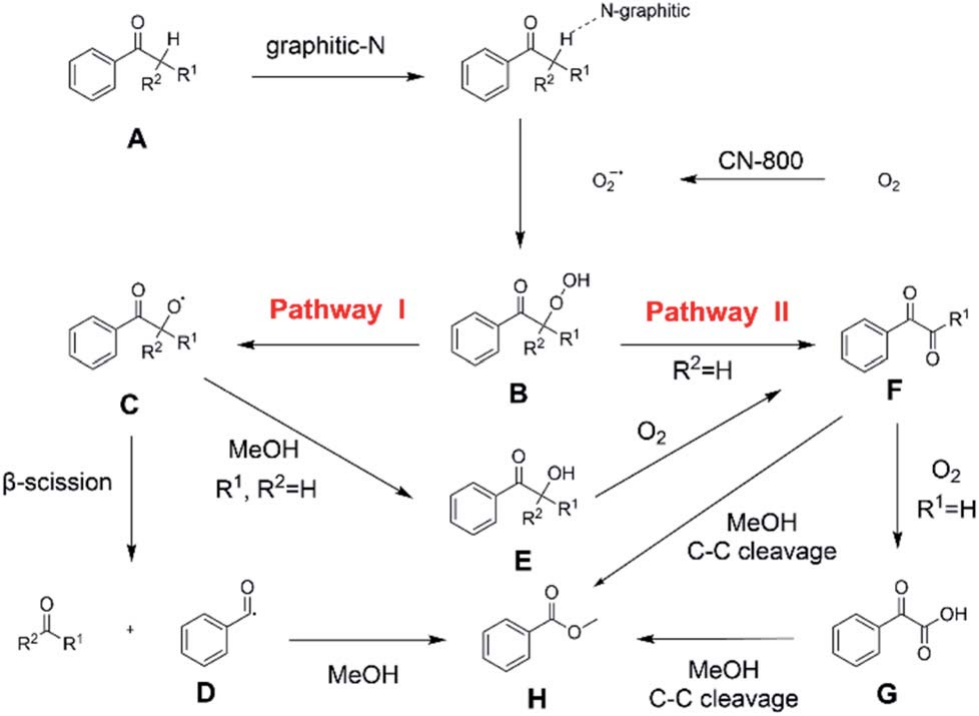
Proposed mechanism of the oxidative cleavage of C–C bonds of ketones to methyl esters over the CN-800.

On the basis of the experimental results above and previous reports, we proposed a plausible mechanism for the aerobic oxidative esterification of ketones into methyl esters *via* C(CO)–C bond cleavage over CN-800 (Scheme 2). Initially, the super oxygen radicals (O_2_˙^−^) were generated by the interaction of O_2_ with the graphitic-N species in CN-800,^56^ and meanwhile, the graphitic-N species with higher electron density could enhance the acidification of the β-C_sp^3^_–H bond in ketones (A). Then, the hydrogen atom in the acidified β-C_sp^3^_–H bond was abstracted by the O_2_˙^−^ radicals to form the hydroperoxide intermediate (B). Finally, the target methyl esters (H) were formed from the intermediate B*via* two potential reaction pathways. In the reaction pathway I, the intermediate B was converted into the hydroxyl radical and β-oxy radical C*via* a homolytic cleavage process. The radical C could be cleaved into the corresponding ketones/aldehydes and benzaldehyde radical D*via* β-scission, and the formed D radical subsequently reacted with MeOH to generate the target methyl esters (H). Alternatively, the radical C could abstract one hydrogen atom from MeOH to generate β-hydroxyl inter-mediate E. When there was one hydrogen atom at the β-C position in the intermediate E, E would be further oxidized into the intermediate F. Control experiments showed that the intermediate E could be clearly detected when using acetophenone as the reactant, while this intermediate was not detected with isobutyrophenone as the reactant. These results indicated that the formation of E was not predominant in reaction pathway I. In another respect, when there was at least one hydrogen atom at the β-C position in B, the reaction could also be proceeded through pathway II. In this pathway, the intermediate B was firstly transformed into the intermediate F*via* Kornblum–DelaMare rearrangement.^57–59^ Eventually, the potentially generated F was converted into the desired product H through two pathways, including (1) F directly reacted with MeOH to be cleaved into the final product H*via* α-diketone cleavage, and (2) If the R^1^ was the hydrogen atom in the intermediate F, F was further oxidized into benzoylformic acid (G), which was subsequently converted into H*via* C–C bond cleavage.

## Conclusions

In conclusion, porous and ultrathin N-doped carbon nanosheets were successfully prepared from chitosan and melamine. The fabricated materials could be used as metal-free and heterogeneous catalysts to efficiently catalyze the oxidative esterification of ketones *via* C(CO)–C bond cleavage into methyl esters. It was observed that the catalytic activity of the catalysts could be significantly affected by the pyrolysis temperature and the weight ratio of the two precursors, and the catalyst CN-800 showed the best performance for the reactions. Various ketones could be selectively oxidized into the corresponding methyl esters at 130 °C in the absence of any basic additives. Systematic analysis indicated that the graphitic-N species were the active sites, and their content and electron density showed strong impact on the catalytic performance. The highest activity of CN-800 resulted from the highest graphitic-N species content and highest electron density, as well as its highest surface area. This work described the first example of metal-free catalytic oxidative esterification of ketones to synthesize esters. We believe that the prepared CN-800 has great potential for application in the synthesis of esters from oxidative conversion of ketones, and the discovery of this work is helpful for designing efficient metal-free catalysts for some other reactions.

## Data availability

The authors declare that all data supporting the findings of this study are available within the paper and its ESI.†

## Author contributions

M. L. H., J. L. S. and B. X. H. proposed the project, designed the experiments, and wrote the manuscript. M. L. H. performed the whole experiments. X. H., H. L. F., T. B. W., Q. L. M. and Z. R. Z performed the analysis of experimental data. J. L. S. and B. X. H. co-supervised the whole project. All authors discussed the results and commented on the manuscript.

## Conflicts of interest

There are no conflicts to declare.

## Supplementary Material

SC-013-D2SC00579D-s001
