# Supplementary material for: Highly efficient C(CO)–C(alkyl) bond cleavage in ketones to access esters over ultrathin N-doped carbon nanosheets
Source: Chem Sci. 2022 Apr 11;13(18):5196–204. doi: 10.1039/d2sc00579d (PMC9093174; doi:10.1039/d2sc00579d)
Supplement: SC-013-D2SC00579D-s001 [file SC-013-D2SC00579D-s001.pdf]

Electronic Supplementary Material (ESI) for Chemical Science.
This journal is © The Royal Society of Chemistry 2022

Supporting Information

Highly Efficient Aerobic Oxidative Esterification of Ketones to Esters over Porous and Ultrathin N-Doped Carbon Nanosheets

Manli Hua,[a,b] Jinliang Song,*[c] Xin Huang,[a,b] Honglei Fan,[a] Tianbin Wu,[a] Qinglei Meng,[a] Zhanrong Zhang[a] and Buxing Han*[a,b]

[a]Beijing National Laboratory for Molecular Science, CAS Key Laboratory of Colloid and Interface and Thermodynamics, CAS Research/Education Center for Excellence in Molecular Sciences, Institute of Chemistry, Chinese Academy of Sciences, Beijing 100190, China. E-mail: hanbx@iccas.ac.cn

[b]School of Chemistry and Chemical Engineering, University of Chinese Academy of Sciences, Beijing 100049, China.

[c]School of Chemical Engineering and Light Industry, Guangdong University of Technology, Guangzhou 510006, China. E-mail: songjl_2021@gdut.edu.cn

Experimental Section

Materials. All commercially available reagents and solvents were used without any further purification. Methanol (AR, 99.5%) and dimethyl sulfoxide (99.9%) were provided by Beijing Analysis Instrument Factory. 2-Acetylthiophene (98%), isobutyrophenone (97%), 3',4',5'-trimethoxyacetophenone (98+%), 4-acetylbiphenyl (98%), 4'-phenoxyacetophenone (98+%), 2',4'-dimethoxyacetophenone (98%), 4'-ethoxyacetophenone (99%), 1'-acetonaphthone (97%), 3',4'-dimethoxyacetophenone (98+%), 1-phenyl-1,2-propanedione (98%), benzyl 4-chlorophenyl ketone (98+%), cyclopentyl phenyl ketone (97%), 1-(4-methoxyphenyl)-2-phenylethanone (98%), 4'-methoxyacetophenone (98%), benzoylformic acid (98%), 2-phenylacetophenone (98%), propiophenone (99%), 2,2-dimethylpropiophenone (97%), 2-hydroxyacetophenone (97+%), 4'-(trifluoromethyl) acetophenone (98%) and 2-phenoxyacetophenone (98%) were purchased from Sigma-Aldrich, Aladdin or Acros. Acetophenone (99%), 2'-methylacetophenone (98%), 3',4'-dimethylacetophenone (98%), butyrophenone (99%), 1,3-indandione (97%), 2-oxo-2-phenylacetaldehyde (95+%), 1-indandione (97%), 2-acetylfuran (99%), 2'-bromoacetophenone (98%), 4'-bromoacetophenone (98%), 3'-bromoacetophenone (97%), 3'-methylacetophenone (98%), 4'-methylacetophenone (98%), 4'-chloroacetophenone (99%), 4'-fluoroacetophenone (98%), 3-acetylpyridine (98%), 4-acetylbenzonitrile (98%), benzaldehyde (99%), benzoic acid (99.5%), 2,6-*di*-*tert*-butyl-4-methylphenol (99%), 1,4-benzoquinone (99%), furfuryl alcohol (98%), ethylbenzene (99.8%), chitosan and melamine were supplied by Beijing InnoChem Science & Technology Co., Ltd.

Preparation of CN-*X*. In a typical procedure, chitosan (3 g) was mixed with melamine (6 g) by ball milling. The obtained solid was calcined at different temperatures (*i.e.*, 800 ^{o}C, 700 ^{o}C, 600 ^{o}C, and 500 ^{o}C) for 5 h under Ar atmosphere. After cooling to room temperature, the material was used directly without any further post-processing. The obtained catalysts were donated as CN-*X* (*X* = pyrolysis temperature). Additionally, it should be pointed out that the ratio of chitosan and melamine was based on the substrate weight.

Preparation of CN-*Chit*. In a typical procedure, chitosan (3 g) was calcined at 800 ^{o}C for 5 h under Ar atmosphere. After cooling to room temperature, the material was used directly without any further post-processing. The obtained catalyst was donated as CN-*Chit*.

Preparation of CN-*1:6* and CN-*1:3*. In a typical procedure, chitosan (2 g or 1 g) was mixed with melamine (6 g) by ball milling. The obtained solid was calcined at 800 ^{o}C for 5 h under Ar atmosphere. After cooling to room temperature, the materials were used directly without any further post-processing. The obtained catalysts were donated as CN-*1:6* or CN-*1:3* based on the mass ratio of chitosan and melamine.

Characterization. Powder X-ray diffraction (PXRD) patterns were performed by Rigaku D/max-2500 X-ray diffractometer equipped with Cu Kα radiation (λ = 1.5418 Å) at 40 kV and 200 mA. The morphologies of the samples were characterized by SEM microscope (SU-8020) and TEM microscope (JEOL-1011) operated at 100 kV and high-resolution TEM (JEOL-2100F) operated at 200 kV. N_2 adsorption-desorption isotherms were obtained using the Micromeritics ASAP 2020M system. X-ray photoelectron spectroscopy (XPS) spectra were on VG Scientific ESCA Lab 250Xi spectrometer using Al Kα radiation with a 500 μm X-ray spot. The contents of C and N in the catalysts were determined by elemental analysis performed on the FLASH EA1112 analyzer. Elemental distribution mappings were examined using a TEM microscope (JEOL-2100F). Raman spectrawere obtained on a laser confocal Raman spectroscopy (Labram-010, Horiba-JY) employing the Nd: YAG laser wavelength of 633 nm. AFM experiment was performed on a Dimension FASTSCANBIO instrument.

General procedure for Oxidation of Ketones to Esters. In a typical experiment, desired amounts of substrates, catalyst (100 mg) and 3 mL of methanol were charged into the 10 mL stainless steel reactor equipped with a magnetic stirrer and a Teflon coating. After being sealed, O_2 (5 bar) was charged into the reactor, and then the

reaction mixture was placed into a constant-temperature air bath, and was stirred at a desired temperature for indicated desired time. After a certain reaction time, the reactor was cool down to room temperature. After the O_2 was removed, ethylbenzene (0.5 mmol) was added into the reactor as the internal standard. The products were analyzed quantitatively by GC (Agilent 6820) equipped with a flame-ionized detector, and identification of the products was performed by GC-MS (Agilent 5975C-7890A).

Reusability of the CN-*800*. To examine the reusability of the CN-*800*, the catalyst was recovered by centrifugation and washed with methanol and ethanol. After being dried under vacuum at 80 ^{o}C for 12 h, the recovered CN-*800* was reused for the next run.

Supplementary Figures

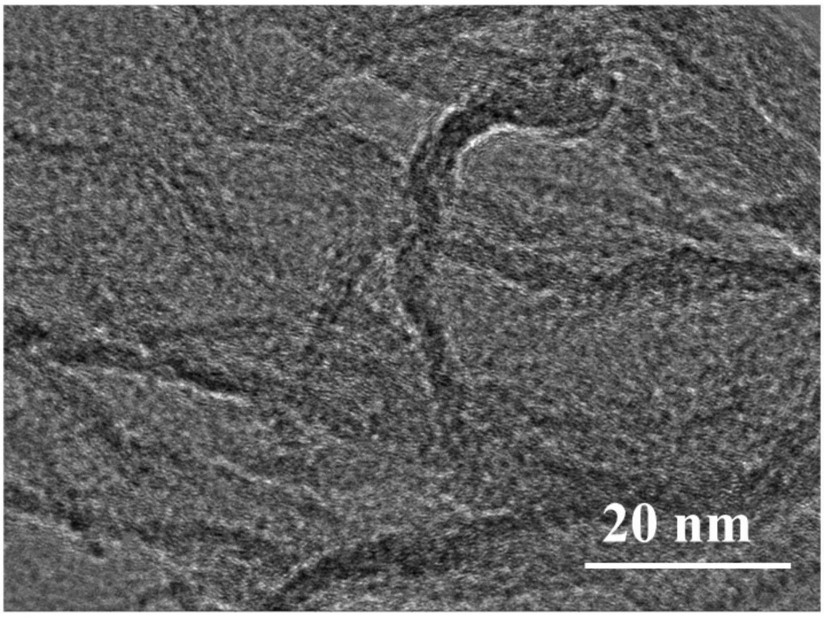

Fig. S1. HR-TEM image of the prepared CN-*800.*

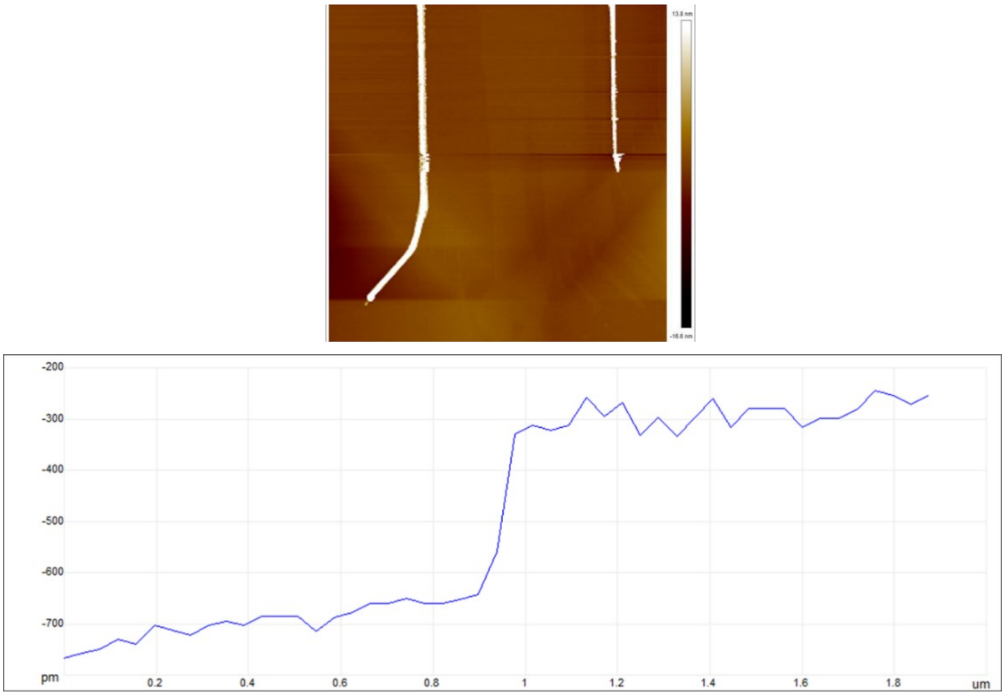

Fig. S2. AFM image of the prepared CN-*800.*

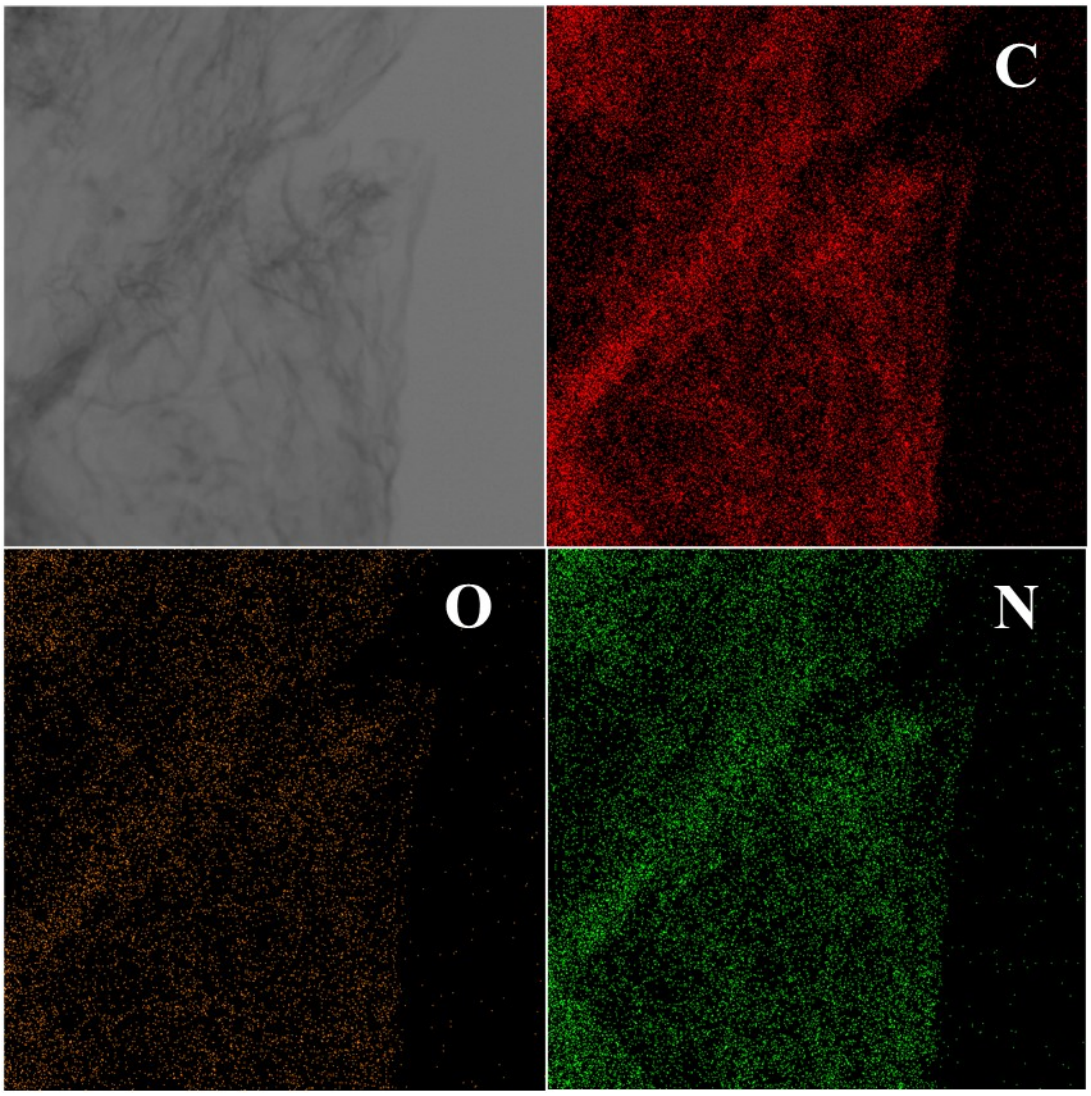

Fig. S3. Elemental mappings of C, N and O of the obtained CN-*800*.

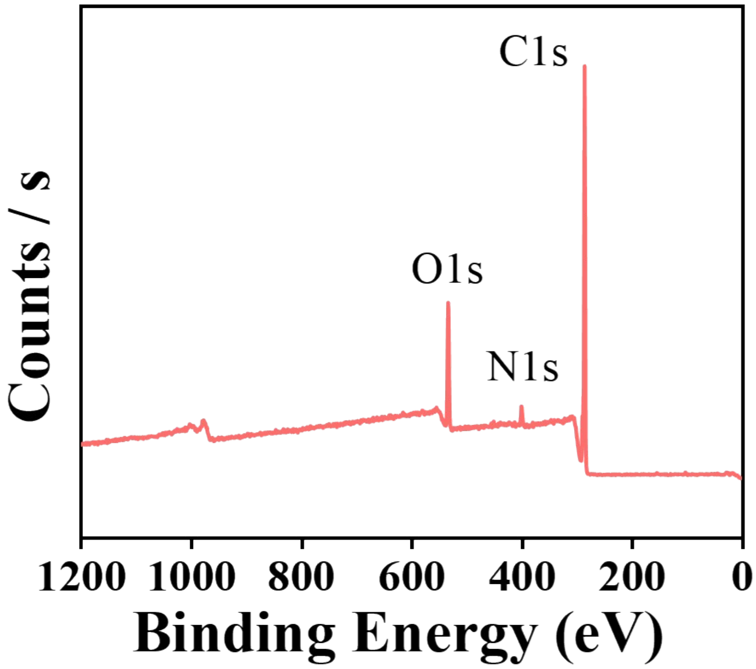

Fig. S4. XPS survey spectrum of CN-*800*.

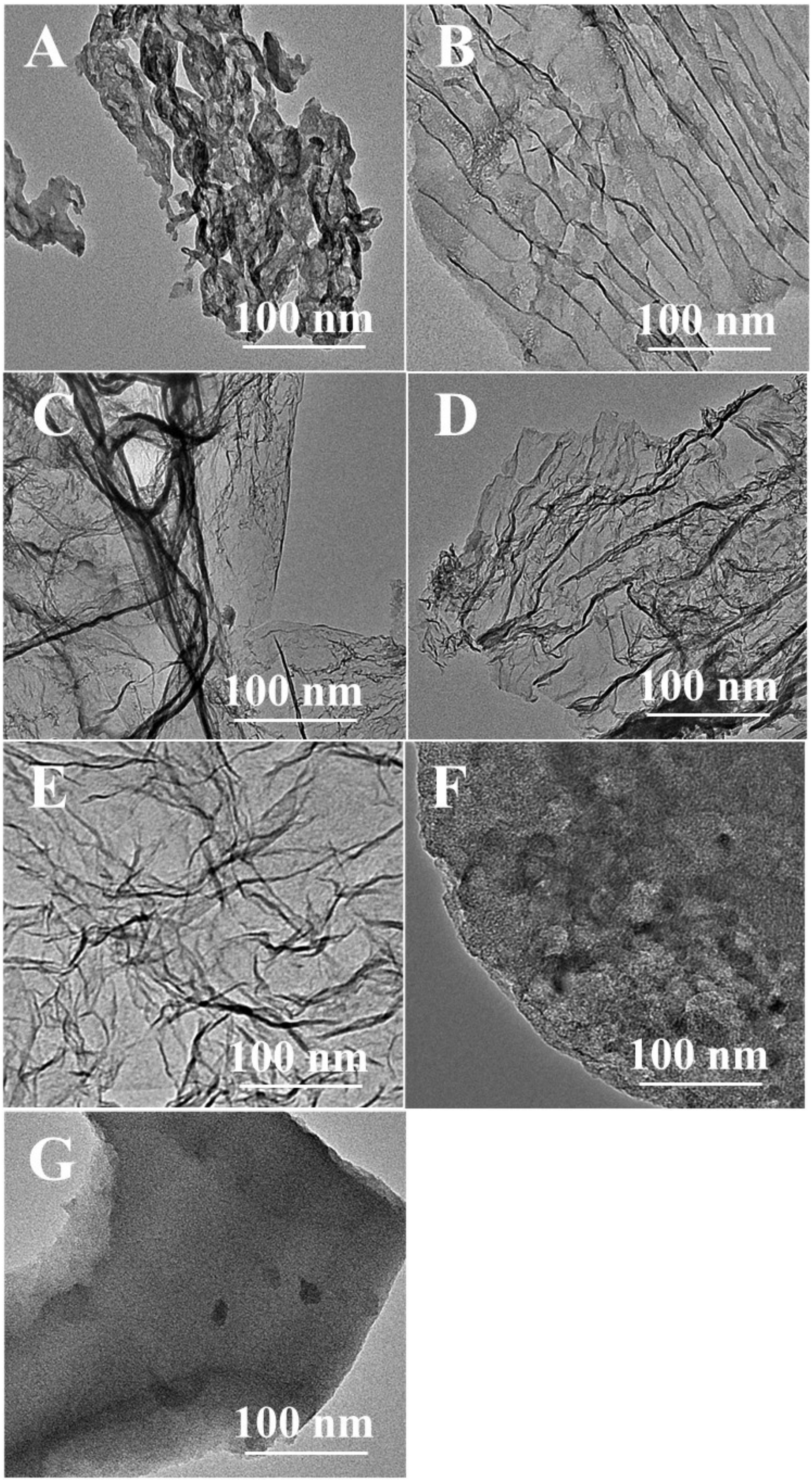

Fig. S5. TEM images of CN-*500* (A), CN-*600* (B), CN-*700* (C), CN-*1:6* (D), CN-*1:3* (E), CN-*Chit* (F), and CN-*Cell* (G).

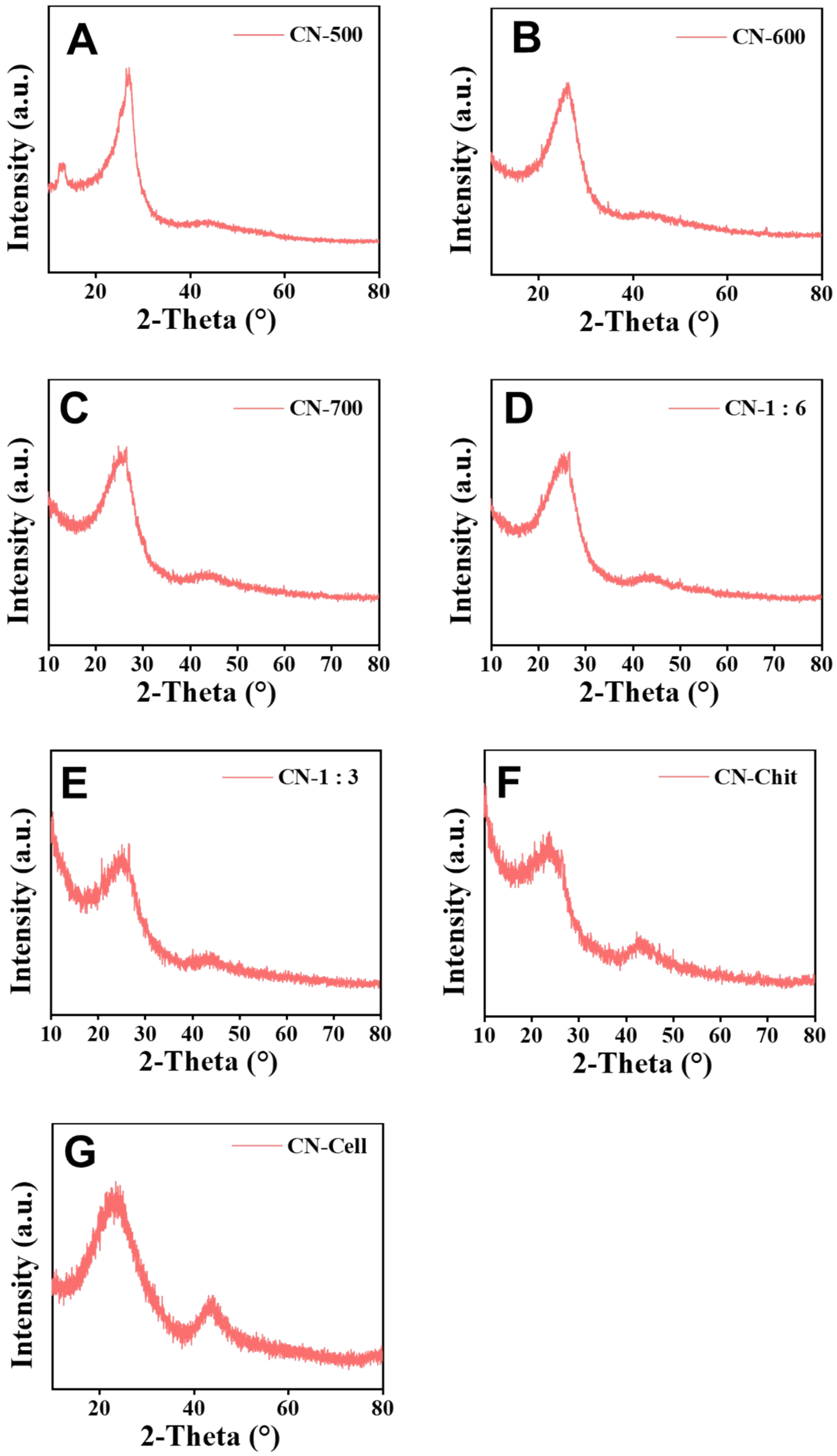

Fig. S6. XRD patterns of CN-*500* (A), CN-*600* (B), CN-*700* (C), CN-*1:6* (D), CN-*1:3* (E), CN-*Chit* (F), and CN-*Cell* (G).

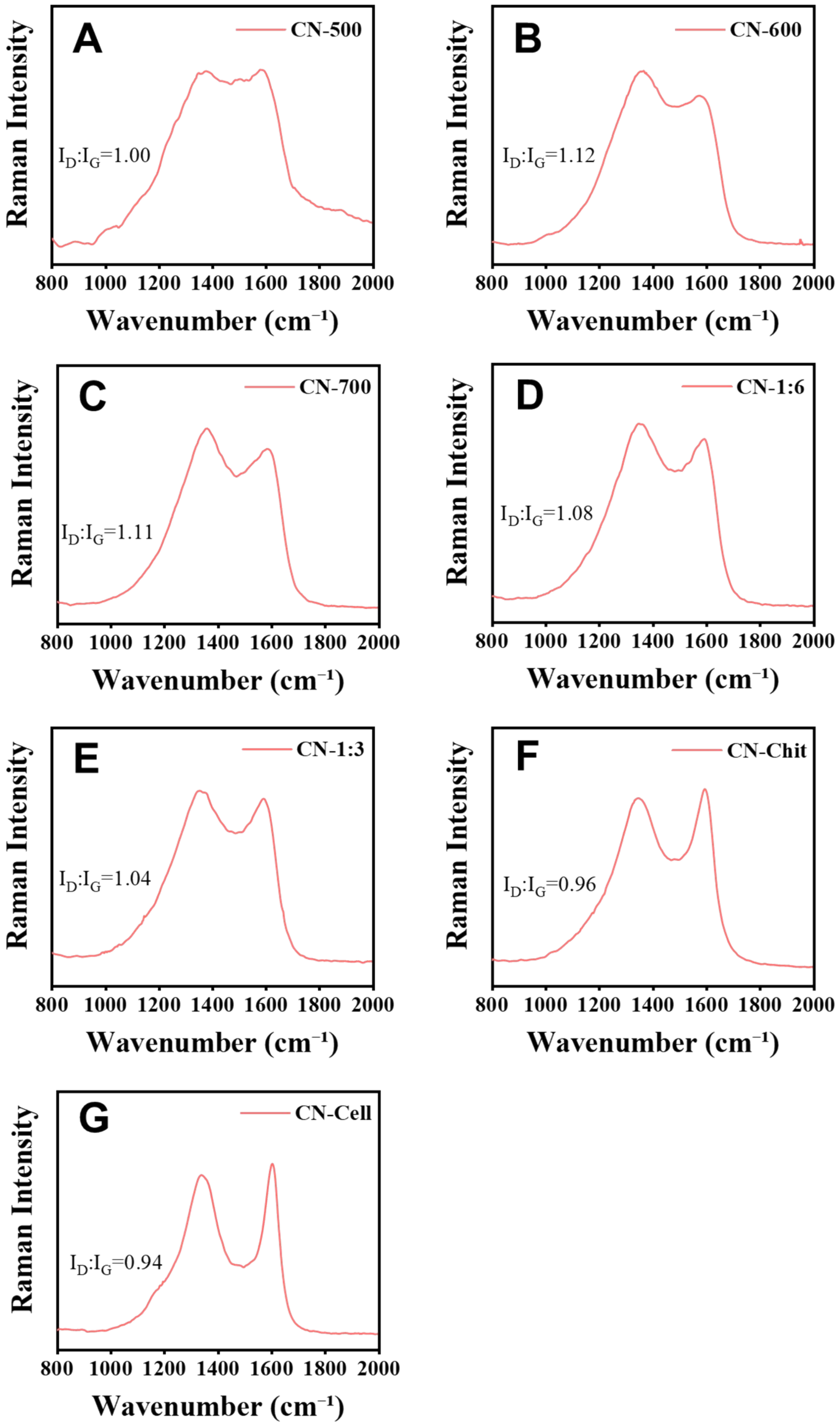

Fig. S7. Raman spectra of CN-*500* (A), CN-*600* (B), CN-*700* (C), CN-*1:6* (D), CN-*1:3* (E), CN-*Chit* (F), and CN-*Cell* (G).

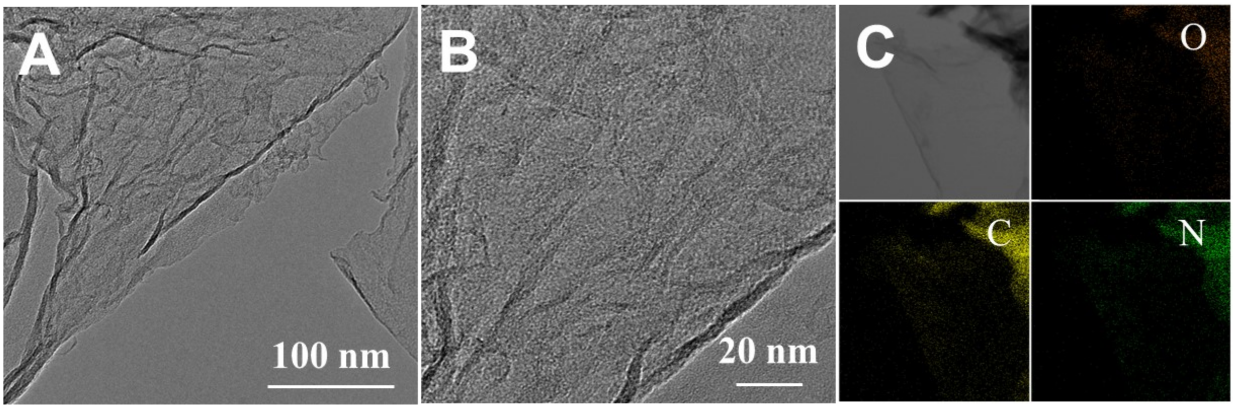

Fig. S8. Characterizations of the recovered CN-*800* after four catalytic cycles. TEM image (A), HR-TEM image (B), and elemental mapping of C, N and O (C).

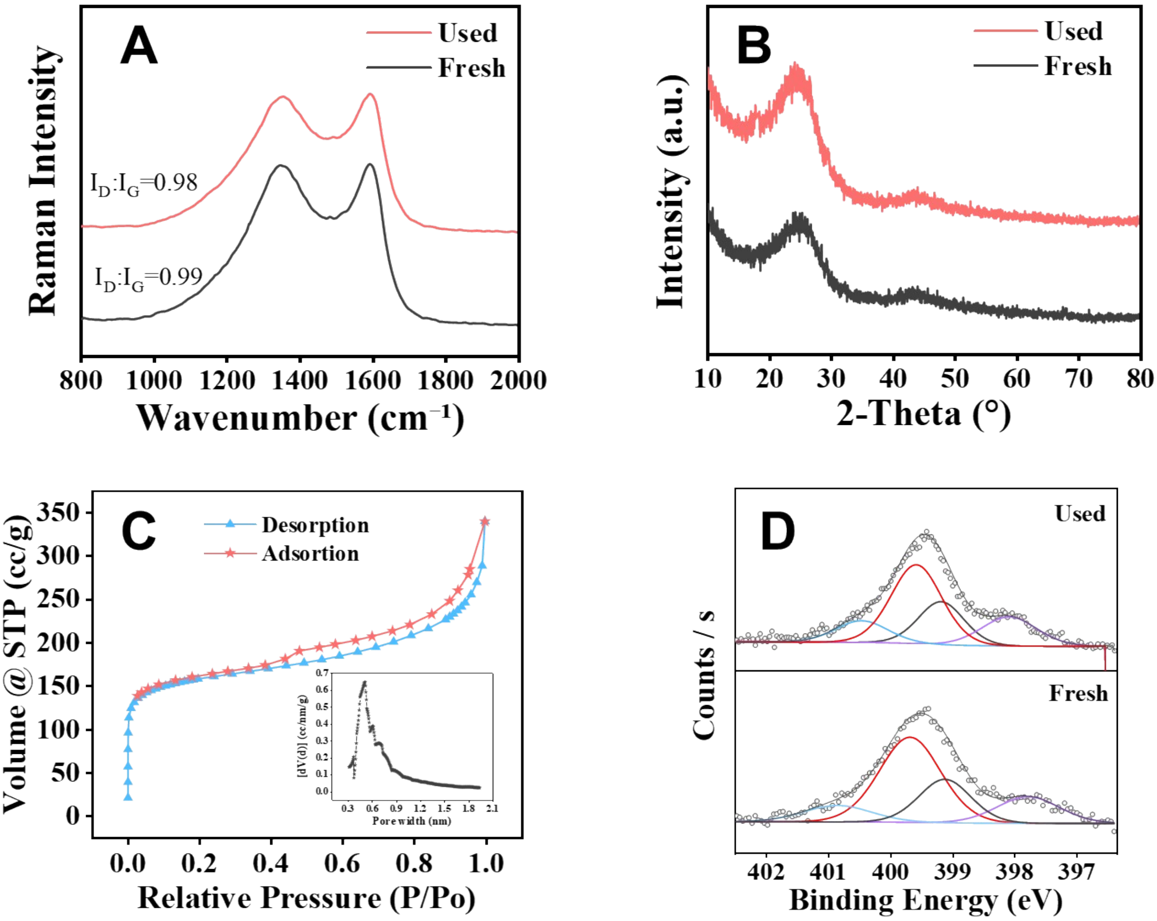

Fig. S9. Characterizations of the recovered CN-*800* after four catalytic cycles. Raman spectra (A), XRD pattern (B), N_2 adsorption-desorption isotherm (C), and XPS spectra of N 1s (D).

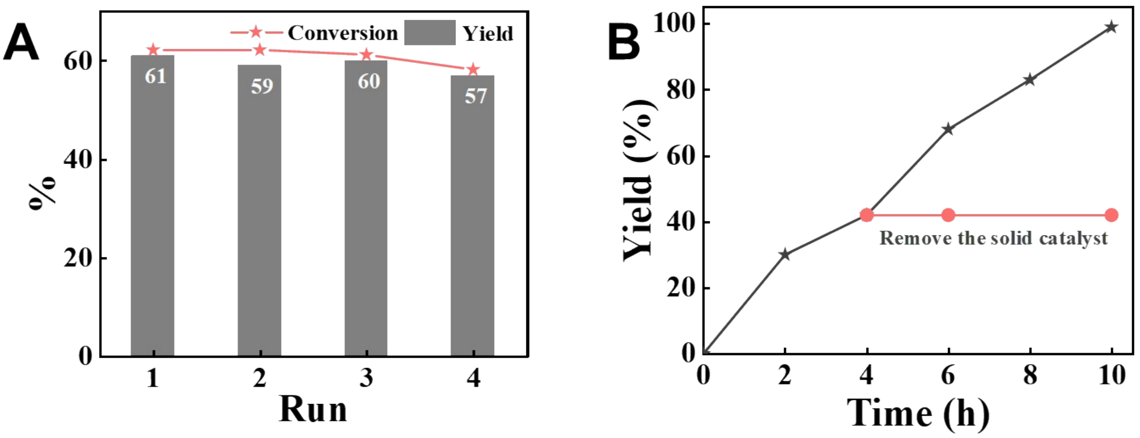

Fig. S10. (A) Reusability of CN-*800*, and (B) Time-yield plots over CN-*800* (black line) or removing CN-*800* after 4 h (red line). Reaction conditions: acetophenone, 0.5 mmol; ethylbenzene, 0.5 mmol; O_2, 5 bar; MeOH, 3 mL; 130 °C; 10 h; usage of CN-*800*, 60 mg for A and 100 mg for B.

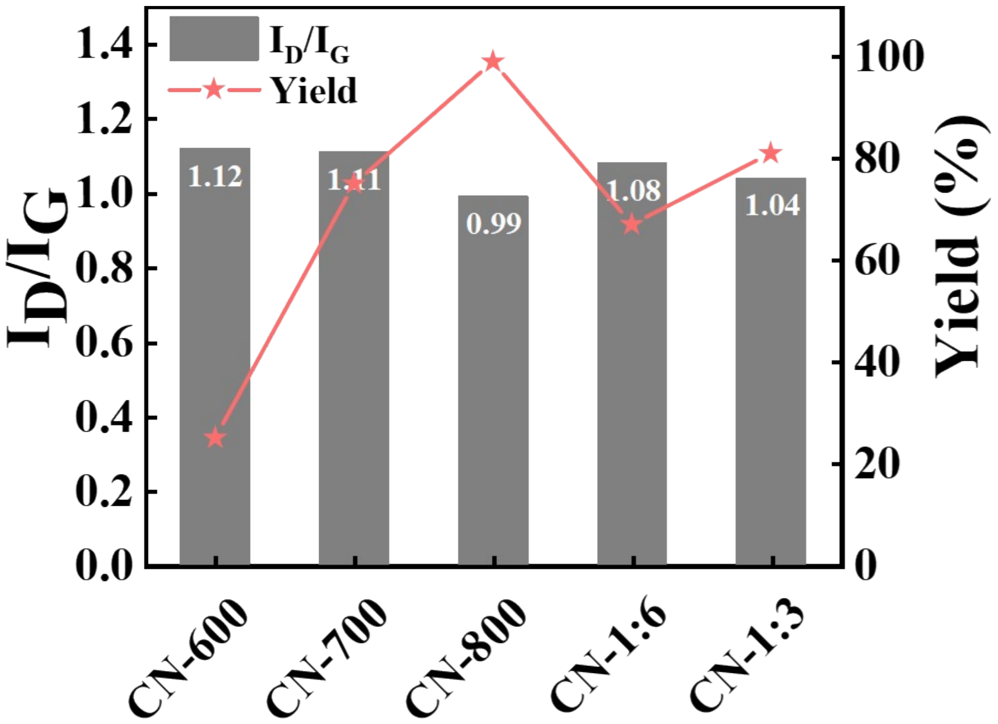

Fig. S11. The correlation between intensity ratio of D/G bands and corresponding reaction efficiency in oxidation cleavage and esterification of acetophenone.

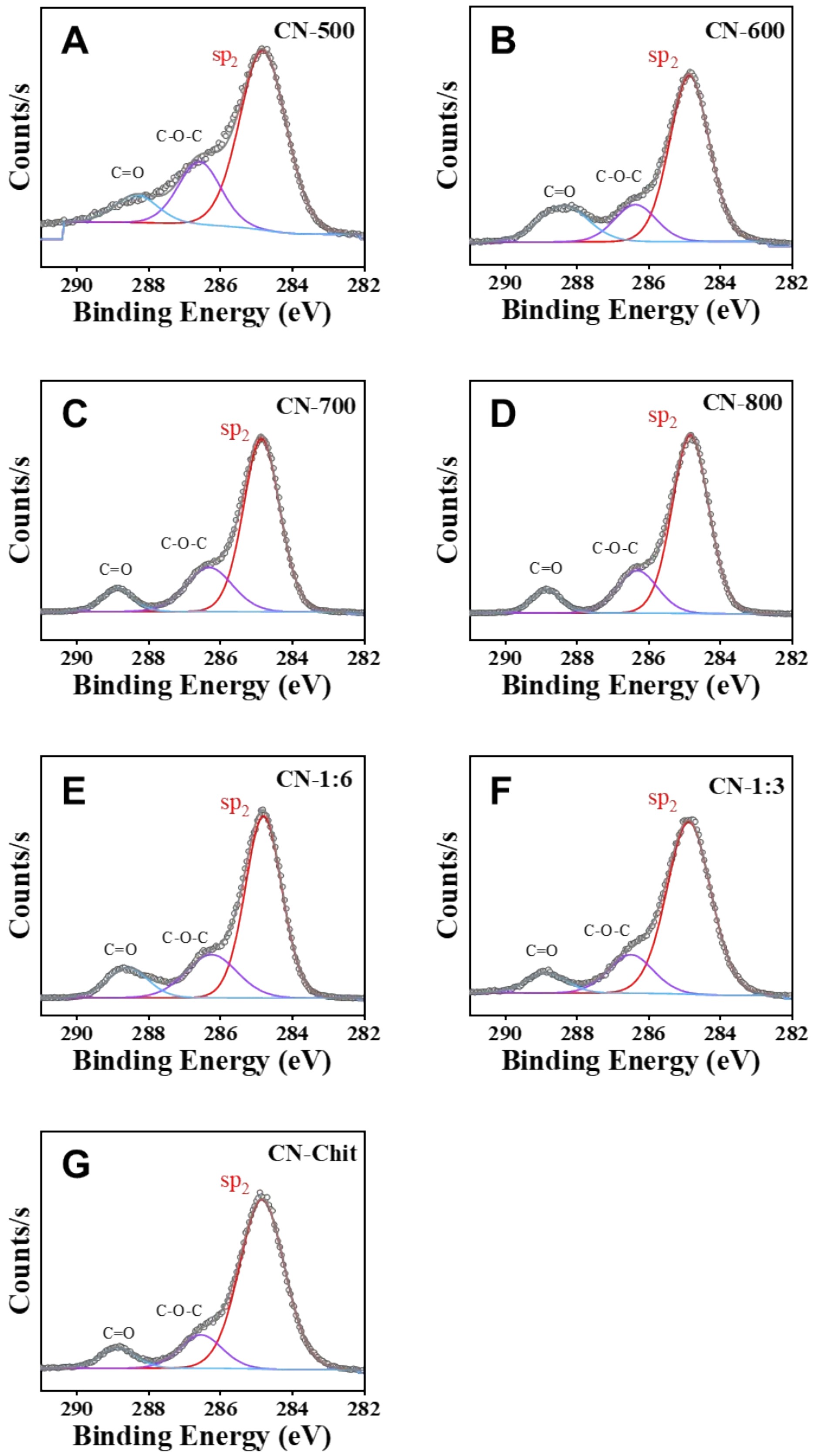

Fig. S12. XPS spectra of C 1s. CN-*500* (A), CN-*600* (B), CN-*700* (C), CN-*800* (D), CN-*1:6* (E), CN-*1:3* (F), and CN-*Chit* (G).

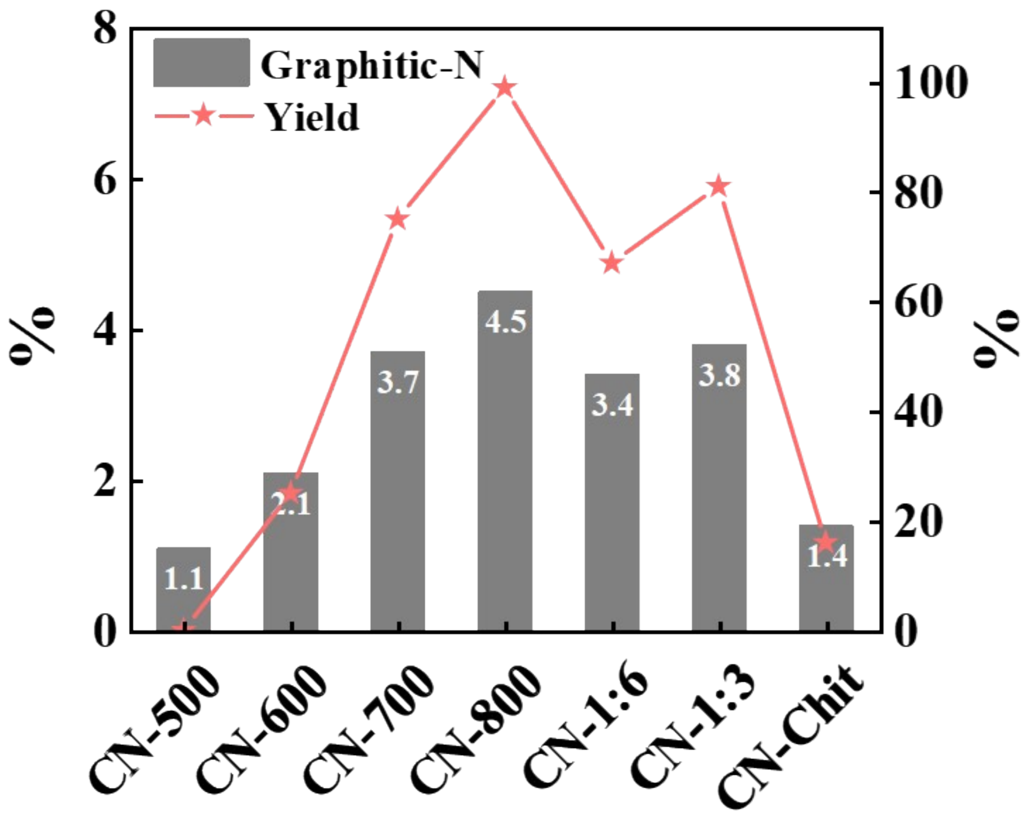

Fig. S13. The correlation between Graphitic-N contents in different catalysts and the corresponding reaction efficiency in oxidation cleavage and esterification of acetophenone.

Supplementary Schemes

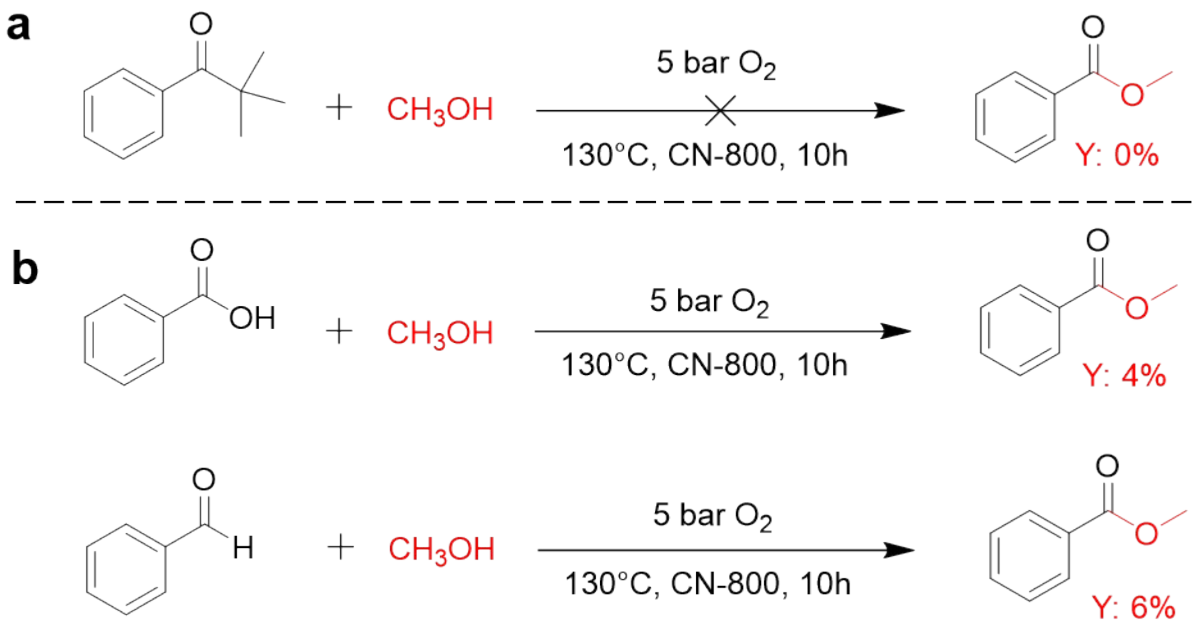

Scheme S1. Conversion of 2,2-dimethylpropiophenone, benzoic acid and benzaldehyde. Reaction conditions: reactant, 0.5 mmol; ethylbenzene, 0.5 mmol; CN-*800*, 100 mg; methanol, 3 mL; O_2, 5 bar; 130 °C; 10 h.

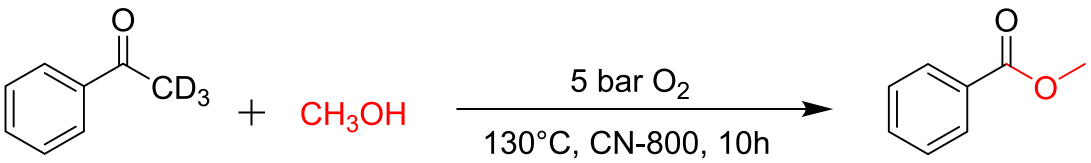

$k_H/k_D=2.35$

Scheme S2. Proton/deuterium kinetic isotope effect of the reaction. Reaction conditions: substrate, 0.5 mmol; ethylbenzene, 0.5 mmol; CN-*800*,100 mg; methanol, 3 mL; O_2, 5 bar; 130 °C; 10 h.

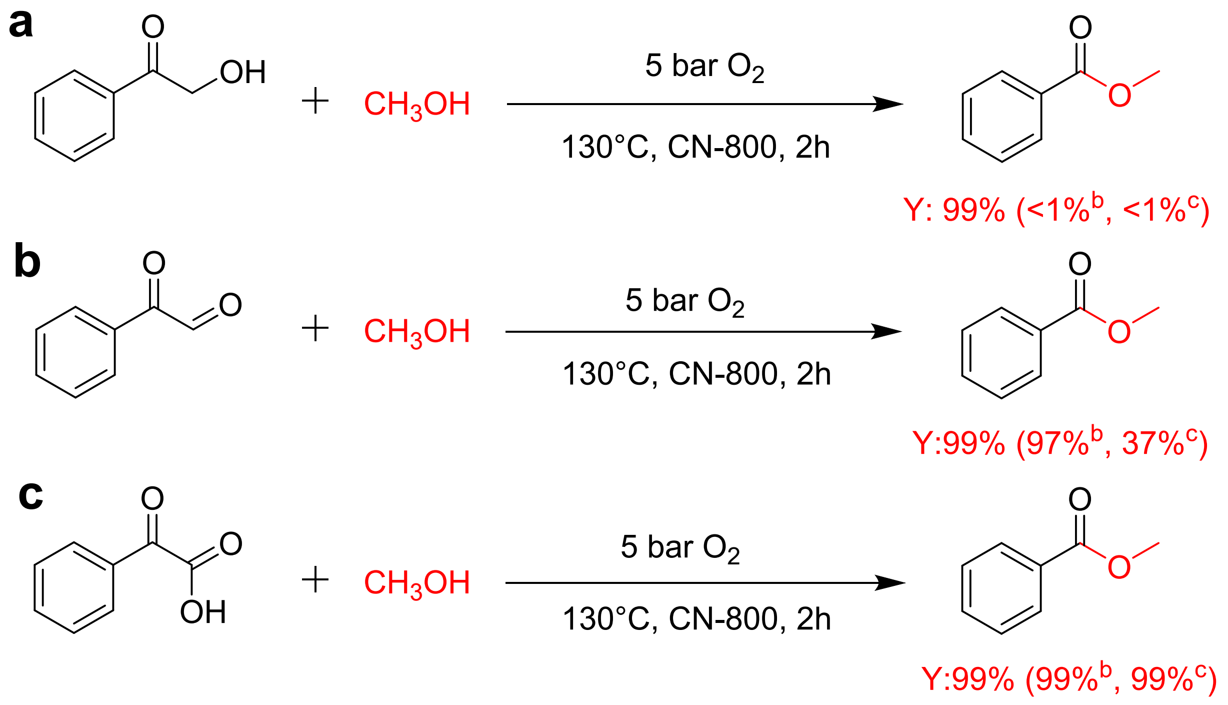

Scheme S3. Conversion of several potential intermediates. Reaction conditions: substrate, 0.5 mmol; ethylbenzene, 0.5 mmol; methanol, 3 mL; O_2, 5 bar; CN-*800*, 100 mg; 130 °C; 2 h. [b]The values in the parentheses were obtained in the presence of benzoquinone (1 mmol). [c]The values in the parentheses were obtained under Ar atmosphere(5 bar) instead of O_2.

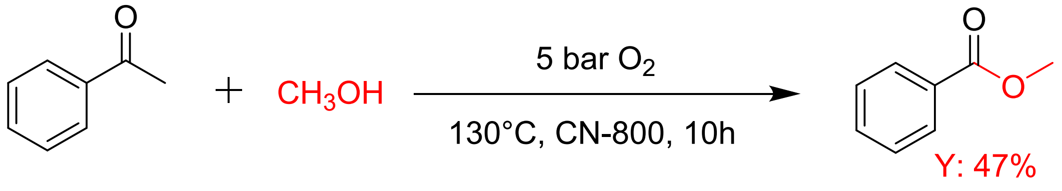

Scheme S4. Conversion of acetophenone in the presence of dimethyl sulfoxide. Reaction conditions: acetophenone, 0.5 mmol; ethylbenzene, 0.5 mmol; methanol, 3 mL; O_2, 5 bar; CN-*800*, 100 mg; dimethyl sulfoxide, 1 mmol; 130 °C; 10 h.

Supplementary Tables

Table S1. BET surface area of the obtained catalysts.

Entry	Catalyst	Surface Area (m^2/g)
1	CN-*500*	14
2	CN-*600*	31
3	CN-*700*	421
4	CN-*Chit*	409
5	CN-*1:6*	481
6	CN-*1:3*	593
7	CN-*800*	631
8[a]	CN-*800*	596
9	CN-*Cell*	312

[a]The recovered CN-*800* after four catalytic cycles.

Table S2. The contents of C, H, N in the catalysts.

Entry	Catalyst	N (%)	C (%)	H (%)
1	CN-*500*	44.9	47.6	1.93
2	CN-*600*	35.45	55.32	1.76
3	CN-*700*	22.32	65.99	1.59
4	CN-*Chit*	5.08	78.4	0.81
5	CN-*1:6*	17.7	68.93	1.26
6	CN-*1:3*	13.04	71.38	1.32
7	CN-*800*	8.72	75.85	1.37
8[a]	CN-*800*	9.11	75.28	1.26

[a]The recovered CN-*800* after four catalytic cycles.

Table S3. Influence of O_2 pressure on the reaction.[a]

Entry	O_2 (bar)	Yield (%)	Conversion (%)
1	1	91	93
2	2	94	95
3	3	98	99
4	4	99	99

[a]Reaction conditions: acetophenone, 0.5 mmol; ethylbenzene, 0.5 mmol; CN-*800*, 100 mg; methanol, 3 mL; 130 °C; 10 h.

Table S4. Chemoselective oxidative cleavage and esterification of C(CO)-C bonds in acetophenone with different aliphatic alcohols.[a]

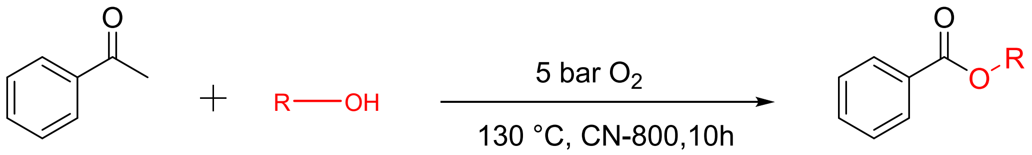

Entry	Aliphatic alcohols	Products	Conversion (%)	Yield (%)
1	Ethanol	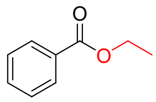	82	38
2	*n*-Propanol	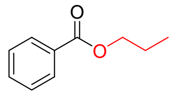	71	18
3	*n*-Butanol	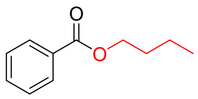	65	3

[a]Reaction conditions: acetophenone, 0.5 mmol; ethylbenzene, 0.5 mmol; CN-*800*, 100 mg; 130 °C; 10 h; K_2CO_3, 0.2mmol; Aliphatic alcohols, 3 mL.

Table S5. The contents of sp^2-C in various catalysts.

Entry	Catalyst	Content of sp^2-C (%)
1	CN-*500*	32.6
2	CN-*600*	37.6
3	CN-*700*	47.5
4	CN-*Chit*	59.6
5	CN-*1:6*	48.9
6	CN-*1:3*	52.1
7	CN-*800*	56.9
